# Short Interdelivery Interval and Neonatal Acid–Base Status, Postpartum Anemia, and Postnatal Depression: A Retrospective Cohort Study with Within-Mother Sensitivity Analysis

**DOI:** 10.3390/jcm15135053

**Published:** 2026-06-29

**Authors:** Ömer Osman Eroğlu, Cansın Eroğlu, Sait Erbey, Mehmet Alican Sapmaz, Murat Polat, Çağanay Soysal

**Affiliations:** Department of Obstetrics and Gynecology, Ankara Etlik City Hospital, Varlık Mahallesi, Halil Sezai Erkut Caddesi, Yenimahalle, 06170 Ankara, Türkiye

**Keywords:** interdelivery interval, neonatal acidosis, postpartum anemia, postpartum depression, Edinburgh Postnatal Depression Scale, birth spacing

## Abstract

**Background/Objectives**: Short interdelivery interval (IDI) is associated with adverse perinatal outcomes, but its relationship with objective neonatal acid–base markers, postpartum anemia, and early postnatal depression remains poorly characterized. We examined IDI < 24 months across four pre-specified outcome domains: neonatal acid–base status, maternal composite morbidity, postpartum anemia, and early postnatal depression. **Methods**: This single-center retrospective cohort study included 851 women with two consecutive singleton live births at Ankara Etlik City Hospital between 2023 and 2025. Women were classified by IDI as short (<24 months; *n* = 635) or standard (≥24 months; *n* = 216). Multivariable logistic regression provided the primary inference. Sensitivity analyses included inverse probability of treatment weighting (IPTW), restricted cubic splines (4 df), within-mother paired analysis, multiple imputation by chained equations (MICE × 20), Firth penalization, E-values, and Benjamini–Hochberg false discovery rate (FDR) correction across 13 outcomes. **Results**: After FDR correction, short IDI was associated with postpartum anemia (adjusted odds ratio [aOR] 1.84, 95% confidence interval (CI) 1.26–2.68; q = 0.022) and with an Edinburgh Postnatal Depression Scale score ≥13 (aOR 1.93, 95% CI 1.20–3.10; q = 0.042); umbilical-artery pH < 7.10 did not cross the FDR threshold (aOR 2.37, 1.17–4.82; q = 0.075) and is reported as an exploratory, hypothesis-generating signal. Postpartum anemia did not mediate the IDI–depression association (proportion mediated 0.6%, 95% CI −13.1% to +13.5%). **Conclusions**: Short IDI was associated with maternal hematologic and psychosocial signals; the IDI–depression link appears non-hematologic. Neonatal acid–base findings did not meet the FDR threshold and are exploratory. Multicenter validation is warranted.

## 1. Introduction

Birth spacing is a modifiable determinant of reproductive health, and its association with maternal and perinatal outcomes has been studied for decades. A landmark meta-analysis of 67 observational studies reported that an interpregnancy interval (IPI: the interval from a previous delivery to conception of the subsequent pregnancy) of less than 6 months, compared with a reference interval of 18–23 months, was associated with increased risks of preterm birth (pooled adjusted odds ratio [OR] 1.40, 95% confidence interval [CI] 1.24–1.58), low birth weight (LBW) (1.61, 95% CI 1.39–1.86), and small-for-gestational-age birth (1.26, 95% CI 1.18–1.33) [1]. This pooled finding is consistent with earlier population-based cohort studies that first established the U-shaped IPI–outcome relationship: Zhu et al. (1999) [2], in a Utah cohort of 173,205 singleton births, demonstrated that an IPI of 18–23 months minimized the risk of low birth weight, preterm birth, and small size for gestational age, with risks increasing progressively at both shorter and longer intervals; Shachar et al. (2016) [3], in a California cohort of nearly one million women, confirmed that IPI < 6 months was associated with a 1.71-fold increase in preterm birth risk (95% CI 1.65–1.78) compared with the 18–23-month reference. Building on this evidence, the World Health Organization (WHO) Technical Consultation on Birth Spacing (Geneva 2005; published 2007) recommended waiting at least 24 months after a live birth before attempting the next pregnancy, and identified birth-to-pregnancy intervals shorter than 18 months as intervals to be avoided [4]. This recommendation remains a cornerstone of current international guidance, and the 24-month threshold continues to serve as an operational decision point in clinical practice.

Two related but non-equivalent measures appear in the international literature. Interpregnancy interval (IPI) refers to the time from a previous delivery to the start of the subsequent pregnancy, while the interbirth or interdelivery interval (IDI) measures the total time between two consecutive deliveries and is approximately equal to IPI plus the gestational duration of the subsequent pregnancy [5]. The two measures are commonly used in this literature, with IPI more frequent in large birth-cohort registries and IDI in record-based clinical studies [5,6]. The present cohort uses IDI as the operational primary exposure, derived from directly source-documented delivery dates in a single-institution electronic health record, in alignment with the WHO 24-month threshold [4].

Despite this strong epidemiologic foundation, three knowledge gaps remain. First, neonatal outcomes in the short birth spacing literature have traditionally been evaluated using phenotypic endpoints such as gestational age, birth weight, or neonatal mortality; the 2019 systematic review by Hutcheon et al. explicitly documented that umbilical artery (UA) pH—a direct biochemical marker of intrapartum fetoplacental oxygenation—was not reported as a primary biomarker in the IDI literature [7]. Second, the same systematic review found no studies in high-income settings reporting postpartum anemia, maternal depression, or maternal mortality in relation to short IPI [7], leaving the maternal evidence base substantially incomplete in two clinically important domains (postpartum hematologic recovery and early depressive symptoms). Third, because much of the existing literature includes high-risk obstetric phenotypes such as preterm birth, low birth weight, and previous perinatal loss, it remains uncertain whether the effect of short IDI persists once these phenotypes are excluded a priori; in the Scottish cohort (*n* = 89,143), the association between short IPI and intrauterine growth restriction disappeared, whereas the associations with preterm birth and neonatal death persisted even among women whose first delivery had been term and uncomplicated [8].

To address these three knowledge gaps simultaneously, we conducted a single-center retrospective cohort study in which high-risk obstetric phenotypes were excluded a priori and evaluated the association of short birth spacing with neonatal acid–base status, maternal hematologic recovery, maternal composite morbidity, and early postnatal depressive symptoms. The pre-specified primary outcomes were UA pH < 7.10 on the neonatal side and a maternal composite morbidity (MCM) outcome, defined as blood transfusion and/or maternal intensive care unit admission within 24 h postpartum. MCM is a pragmatic two-component composite operationalized for the present cohort and is narrower in scope than the multi-indicator Severe Maternal Morbidity (SMM) framework defined by the U.S. Centers for Disease Control and Prevention [9] and the WHO maternal near-miss criteria, which identify cases of life-threatening obstetric complications by organ-system dysfunction [10,11]; the rationale and operational distinctions are detailed in Section 2.4.2. Key secondary outcomes included postpartum anemia (hemoglobin < 10 g/dL at 24 h) and an Edinburgh Postnatal Depression Scale (EPDS) score of ≥13. We tested the hypothesis that short IDI (<24 months) would be associated with adverse signals across these four outcome domains independently of high-risk obstetric phenotypes. The detailed analytic strategy—including the within-mother sensitivity analysis, restricted cubic spline modeling of continuous IDI, and the multilayered robustness framework—is described in the Methods section (Section 2.6).

## 2. Materials and Methods

### 2.1. Study Design and Setting

This single-center retrospective cohort study was conducted at the Department of Obstetrics and Gynecology, Ankara Etlik City Hospital, Ministry of Health, Ankara, Türkiye. Ankara Etlik City Hospital is a public tertiary referral center for high-risk pregnancies in the capital of Türkiye, with an annual delivery volume of approximately 8000 births, integrated regional perinatology services, a level III neonatal intensive care unit, and adult intensive care facilities. The hospital opened in February 2022, and all obstetric and neonatal records have been maintained in a single uninterrupted electronic health record (EHR) system since that time. The source population consisted of women with two consecutive singleton live births documented at this center between 1 January 2023 and 31 December 2025. Data were obtained retrospectively from the hospital EHR system, labor ward and neonatal intensive care unit records, the laboratory information system (LIS), and postpartum outpatient follow-up records. Data extraction was performed independently by two investigators using a pre-defined standardized data dictionary, and discrepancies were resolved by consensus after verification against the source records. Reporting followed the Strength-ening the Reporting of Observational Studies in Epidemiology (STROBE) statement [12].

### 2.2. Participants

#### 2.2.1. Study Population and Paired Within-Mother Data Structure

The analytic cohort comprised women with two consecutive singleton live births at Ankara Etlik City Hospital between 1 January 2023 and 31 December 2025. Because the hospital opened in February 2022, the source population was structurally limited to this period; women whose earlier birth had occurred at another institution were not included, as clinical data for the previous birth were not available in our system. This design eliminates one of the most common measurement limitations in the IDI literature: reliance on self-reported information or heterogeneous external records for the previous birth.

The unit of analysis was a paired delivery record for each woman: 851 women × 2 deliveries = 1702 delivery records. For each woman, the first birth (the earlier documented birth at the same hospital) and the index birth (the second birth) were recorded as separate entries in the dataset; the exposure, interdelivery interval (IDI), was then calculated once at the woman level as the interval between these two delivery dates. This design supported two complementary analytic approaches: (i) cohort-level comparisons between short and standard IDI groups, and (ii) within-mother comparisons based on the paired structure of two consecutive births in the same woman (see Section 2.6.3 for details).

Because both births occurred within the same 3-year window, the interval between them fell within an approximate 10–36-month range, allowing meaningful separation between short IDI (<24 months) and standard IDI (≥24 months) categories and strengthening the operational validity of the WHO 24-month threshold. In addition, both births took place within the same institution under the same clinical protocols, laboratory workflows, and equipment, which minimized design-level sources of bias such as era drift and inter-center heterogeneity.

#### 2.2.2. Inclusion Criteria

- Two consecutive singleton live births at Ankara Etlik City Hospital between 1 January 2023 and 31 December 2025.
- Electronic verification of the dates of both births.
- Reliable gestational age estimation for both births, based on first-trimester ultrasound confirmation and/or last menstrual period.
- Documentation of the primary and secondary outcome variables for both births in the hospital records.

#### 2.2.3. Exclusion Criteria

- Any multiple gestation (twins, triplets, or higher-order multiples).
- Any delivery occurring before 24 completed gestational weeks.
- Antepartum fetal death in either birth.
- Major congenital anomaly in either birth.
- Hypertensive disorders of pregnancy (gestational hypertension, preeclampsia, eclampsia, or chronic hypertension with or without superimposed preeclampsia) in either birth.
- Gestational diabetes mellitus or pre-gestational diabetes requiring pharmacologic management in either birth.
- Placenta previa or placenta accreta spectrum disorders in either birth.
- Fetal growth restriction requiring perinatology follow-up in either birth.
- Records in which IDI could not be calculated.
- Records with ≥50% missingness in the core analytic variables required for modeling (exposure, primary outcomes, and adjustment variables).

Exclusion criteria were applied through clinical adjudication of the full patient epicrisis (discharge summary and delivery narrative) by two obstetricians (blinded to exposure status), rather than relying on ICD-10 codes alone, because ICD-10 coding of obstetric comorbidities in the institutional electronic health record was incomplete for a substantial subset of records.

**Figure 1:**
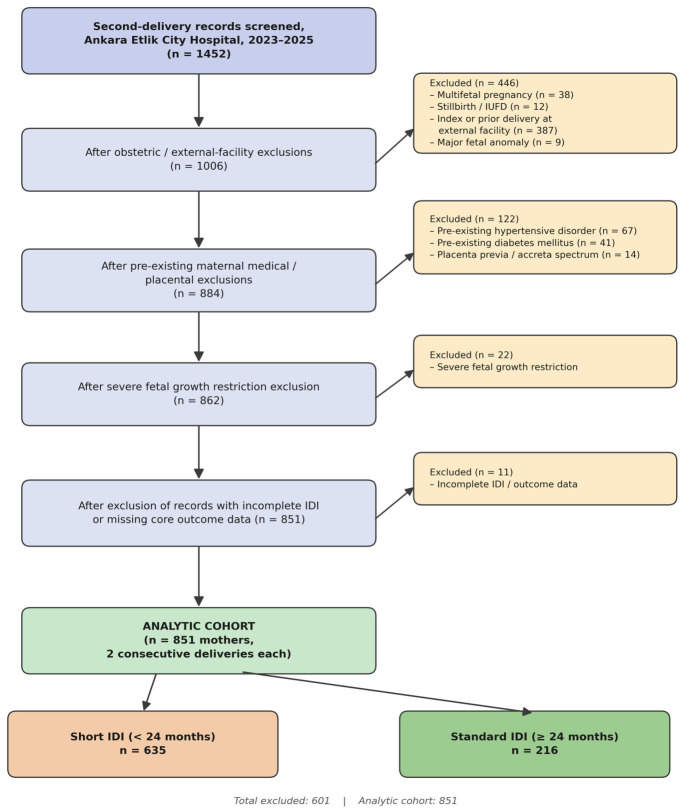
Cohort flow (STROBE). Screening, eligibility, exclusions, and analytic sample. Of 1452 second-delivery records screened, 851 women (Short IDI < 24 months, *n* = 635; Standard IDI ≥ 24 months, *n* = 216) met all eligibility criteria and formed the primary analytic cohort with 1702 paired deliveries (each woman contributing one previous and one index delivery at the same institution). The interdelivery interval was computed from the previous live-birth date to the index (second) delivery.

The final analytic cohort consisted of 851 women, including 635 with short IDI (<24 months) and 216 with standard IDI (≥24 months). The participant flow diagram and record-level exclusions are presented in Figure 1.

### 2.3. Exposure Definition

#### 2.3.1. Operational Definition and Measurement

In this study, the interdelivery interval (IDI) was selected as the primary exposure measure, defined as the time between two consecutive deliveries (Introduction; conceptual framework summarized in Section 1, second paragraph). The relationship to the alternative interpregnancy interval (IPI) measure is given by IDI = IPI + gestational duration of the index pregnancy.

IDI was calculated as the number of days between the index delivery date and the previous live-birth date, then converted into months for analysis (1 month = 30.4375 days). The definition of a previous live birth followed WHO criteria and included all births at or beyond 22 completed gestational weeks with signs of life after delivery; miscarriages (<22 weeks) and elective terminations were not counted as previous births. Because both delivery dates used to define IDI were source-documented in the same hospital electronic health record (EHR), the exposure was ascertained with low measurement error—eliminating both recall bias for the previous birth and heterogeneity of external records that affect conventional retrospective designs.

#### 2.3.2. Threshold Selection and Sensitivity Analyses

For the primary analysis, IDI was categorized as short IDI (<24 months) and standard IDI (≥24 months), consistent with the WHO recommendation [4] and corresponding to the biologically meaningful interval for critical maternal recovery processes (replenishment of iron stores and uterine involution). The a priori primary comparison of the study was based on this dichotomous classification.

To assess dependence on the chosen cutoff, two complementary pre-specified approaches were used: (a) IDI in months was modeled as a continuous variable using restricted cubic splines (RCS; 4 degrees of freedom), with 24 months (730 days) as the reference knot; and (b) separate logistic regression models were run using alternative cutoffs (6, 12, and 18 months) and the resulting effect estimates were compared. These analyses are reported in Supplementary Table S1.

### 2.4. Outcome Definitions

#### 2.4.1. Primary Neonatal Outcome

The primary neonatal outcome was clinically meaningful fetal acidemia, defined as an umbilical artery (UA) pH < 7.10. Umbilical arterial blood gas samples were obtained immediately after delivery from a clamped cord segment, placed in a heparinized syringe, and analyzed within 20 min using a RAPIDPoint 500 blood gas analyzer (Siemens Healthcare Diagnostics Inc., Erlangen, Germany) in the delivery ward. The same standard was applied to both the previous and index births.

#### 2.4.2. Primary Maternal Outcome: Maternal Composite Morbidity (MCM)

The primary maternal outcome was maternal composite morbidity (MCM), pre-specified as a composite of peripartum blood transfusion and/or maternal intensive care unit (ICU) admission. The composite event was ascertained by cross-linking transfusion and intensive care records with ICD-10-coded hospital data, following pre-defined institutional protocols.

Terminology note: MCM is a pragmatic operational definition specific to the present study that combines two clinical events—transfusion and ICU admission—that can be captured completely and directly from our institutional records. MCM is narrower in scope than two widely cited frameworks for severe maternal morbidity. The U.S. Centers for Disease Control and Prevention (CDC)’s Severe Maternal Morbidity (SMM) framework [9] is administrative-data-based and uses ICD diagnosis and procedure codes to identify cases with severe complications during delivery and postpartum hospitalizations; the CDC framework currently includes 25 severe morbidity indicators for delivery hospitalizations and 18 for postpartum hospitalizations. The WHO maternal near-miss criteria [10,11] apply an organ-system-dysfunction-based identification using clinical, laboratory, and management-based markers; a maternal near-miss case is defined as a woman who nearly died but survived a complication that occurred during pregnancy, childbirth, or within 42 days of termination of pregnancy. MCM as defined here captures a subset of these cases—specifically the two intervention-based markers (transfusion and ICU admission) that are reliably documented in our institutional records. To avoid terminological ambiguity, the terms SMM and near-miss are not used as synonyms for MCM in the present manuscript; instead, the abbreviation MCM is applied consistently throughout the text, tables, and Supplementary Materials.

#### 2.4.3. Key Secondary Hematologic Outcome

The key hematologic secondary outcome was postpartum anemia, defined as a hemoglobin (Hb) value < 10 g/dL at 24 h postpartum. Routine complete blood count values obtained 24–28 h after delivery were used. Pre-operative anemia (last pre-delivery Hb < 11 g/dL) was conceptualized as a mediator rather than an adjustment covariate in the primary postpartum anemia model and was therefore excluded from the main adjustment set for this outcome; in a complementary sensitivity model, pre-operative anemia was included for adjustment. The 24 h hemoglobin values for both births were available as separate variables in the dataset, making them suitable for within-mother δ analyses.

#### 2.4.4. Key Secondary Psychosocial Outcome

The key psychosocial secondary outcome was postnatal depression risk, defined as an Edinburgh Postnatal Depression Scale (EPDS) score ≥ 13 at postpartum day 10. The EPDS was administered in person on postpartum day 10 as part of the institutional postpartum follow-up protocol, using the validated Turkish version [13]. The data used in the present study were derived from this routine clinical documentation. Because this practice has been standard since the hospital opened, EPDS scores for both the previous and index births were obtained using the same method.

#### 2.4.5. Exploratory and Safety Outcomes

Pre-specified exploratory outcomes included the following: 5 min Apgar score < 7, neonatal intensive care unit admission, birth weight, preterm birth (<37 weeks), small for gestational age (SGA; <10th percentile), postpartum hemorrhage (estimated blood loss ≥ 1000 mL), blood transfusion, uterine atony, postpartum infection, and continuous EPDS score change. Operational definitions of all primary, key secondary, and exploratory outcomes are provided in Supplementary Table S2.

### 2.5. Covariates and Adjustment Variables

Covariates were selected a priori based on the underlying directed acyclic graph (DAG), constructed with DAGitty (http://www.dagitty.net, accessed on 9 April 2026) and provided as Supplementary Figure S1, together with variables consistently identified as confounders in the prior literature. The DAG depicts IDI as the exposure, four outcome domain nodes (neonatal acid–base status, maternal composite morbidity, postpartum anemia, postpartum depression), adjustment covariates, pre-operative anemia as a mediator, and unmeasured confounders (body-mass index, smoking, socioeconomic status, and breastfeeding duration) as dashed nodes.

Adjustment sets were defined in an outcome-specific manner, in direct correspondence with the causal structure depicted in the DAG. For each primary outcome the minimum sufficient adjustment set (MSAS) was derived from the DAG using the back-door criterion, with the following resulting structure:

(i) For UA pH < 7.10 (neonatal acid–base outcome), the MSAS comprised maternal age, parity, gestational age, pre-operative anemia, mode of delivery, induction, prior cesarean, and migrant status. Pre-operative anemia was included here because it acts as a confounder rather than a mediator for the neonatal acid–base pathway, plausibly through chronic placental oxygen-transport reserve depletion (King 2003 [14]).

(ii) For 24 h postpartum anemia (maternal hematologic outcome), the MSAS comprised maternal age, parity, gestational age, mode of delivery, induction, prior cesarean, and migrant status. Pre-operative anemia was deliberately excluded from this adjustment set because it is the proximate biological mediator on the causal path: short IDI → pre-operative iron depletion → 24 h postpartum anemia; conditioning on a mediator would block the mediated component of the effect (overadjustment bias).

(iii) For EPDS ≥ 13 (psychosocial outcome), the MSAS was reduced to maternal age, parity, mode of delivery, and migrant status, reflecting the a priori DAG in which gestational age and obstetric labor variables are not on the causal pathway from short IDI to psychosocial outcomes assessed at postpartum day 10.

Unmeasured confounders—body-mass index, smoking, socioeconomic status, and breastfeeding duration—were retained as dashed nodes in the DAG and are explicitly addressed in the E-value sensitivity analyses (Section 2.6.9) and in the residual confounding discussion (Section 4.6).

#### 2.5.1. Demographic Covariates

- Maternal age (continuous, years).
- Migrant status (non-Turkish citizen: yes/no; foreign national mother registered within the Turkish health system).

#### 2.5.2. Obstetric History and Previous Birth Phenotype

- Parity (number of previous live births: 1, 2, or ≥3).
- Previous cesarean delivery (yes/no).
- Recorded phenotypic characteristics of the previous birth (e.g., previous preterm birth, previous postpartum hemorrhage, previous pre-operative anemia), which could be incorporated for control purposes in the within-mother analysis (Section 2.6.3).

#### 2.5.3. Current Pregnancy

- Gestational age (GA, weeks, continuous).
- Pre-pregnancy/pre-operative anemia, defined as pre-delivery hemoglobin <11 g/dL (mediator variable; excluded from adjustment in the primary postpartum anemia model).

Gestational age served two distinct causal roles depending on the outcome. For cross-sectional primary outcomes (UA pH, 24 h postpartum hemoglobin, and postpartum day-10 EPDS), GA was treated as a confounder and included in the adjustment set. For secondary outcomes in which GA lies on the causal pathway (preterm birth, SGA, and birth weight), GA was excluded from the adjustment set to avoid overadjustment through conditioning on a mediator. This distinction was documented for each outcome–model pair and is summarized in Supplementary Table S2.

#### 2.5.4. Delivery Management

- Mode of delivery (cesarean vs. vaginal).
- Labor induction (any pharmacologic or mechanical induction: yes/no).

#### 2.5.5. Missing Data Management

Variable-level missingness is reported in Supplementary Table S3a. The primary analysis used a complete-case approach with the pre-specified adjustment set. As a robustness check, multiple imputation by chained equations (MICE) under the missing-at-random (MAR) assumption was implemented as a complementary sensitivity analysis using miceforest (random forest-based imputation; 20 imputed datasets × 10 iterations). Estimates were pooled on the log-OR scale using Rubin’s rules, with the Barnard–Rubin small-sample degrees-of-freedom correction applied [15] (Supplementary Table S4).

### 2.6. Statistical Analysis

**Figure 2:**
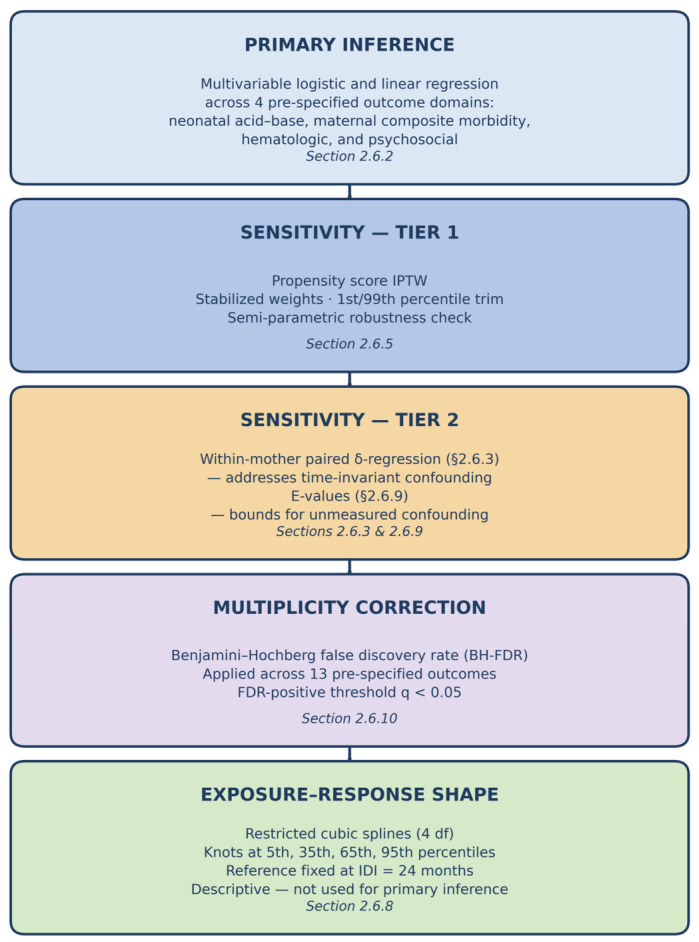
Analytical strategy: hierarchy of inference. Primary inference is drawn from the multivariable regression models. All subsequent analyses serve as sensitivity tiers: Tier 1 (propensity score IPTW) addresses model specification; Tier 2 (within-mother δ-regression and E-values) addresses time-invariant and unmeasured confounding. Multiple-testing correction (BH-FDR) is applied across the 13 pre-specified outcomes. Restricted cubic splines characterize the exposure–response shape and are not used for primary inference.

Analytic framework and hierarchy: The inferential architecture is hierarchical: primary inference is drawn from the multivariable logistic and linear regression models (Section 2.6.2), and complementary sensitivity analyses (Section 2.6.3, Section 2.6.5, Section 2.6.6, Section 2.6.7, Section 2.6.8 and Section 2.6.9) provide robustness checks under different identifying assumptions. The full analytic hierarchy is summarized graphically in Figure 2. Multiple testing across the 13 pre-specified outcomes is controlled by the Benjamini–Hochberg false discovery rate procedure (Section 2.6.10). Causal language is avoided throughout; effect estimates are interpreted as adjusted associations, not as causal effects (Section 4.6, “Causal-language disclaimer”).

#### 2.6.1. Descriptive Statistics

Continuous variables were summarized as mean ± standard deviation when approximately normally distributed, or as median (interquartile range, IQR) when not. Categorical variables were summarized as counts and percentages. Baseline characteristics were compared between the short and standard IDI groups, and standardized mean differences (SMDs) were calculated to identify clinically meaningful imbalance; |SMD| > 0.10 was considered indicative of imbalance.

#### 2.6.2. Primary Analysis: Multivariable Logistic and Linear Regression

The primary inferential framework comprised multivariable logistic regression models for binary primary and secondary outcomes, and multivariable linear regression models for continuous outcomes, using the pre-specified adjustment sets. For crude group comparisons, Student’s *t*-test or the Mann–Whitney U test was used for continuous variables depending on distributional assumptions, while the chi-square test or Fisher’s exact test was used for categorical variables when expected cell counts were <5. Effect estimates were reported as adjusted odds ratios (aORs) or β coefficients with 95% CIs. Model fit was assessed using the Hosmer–Lemeshow goodness-of-fit test (all *p* > 0.05 across the primary outcomes), and multicollinearity was evaluated using variance inflation factors (all VIF < 2.0 for the primary adjustment set). This multivariable framework served as the primary inference engine of the study. Variance inflation factors for all covariates in the primary adjustment set are reported in Supplementary Table S10.

#### 2.6.3. Within-Mother δ-Regression (Sensitivity Analysis)

As a sensitivity analysis within the broader analytic hierarchy (see preamble), a within-mother δ-regression framework was applied, in which each woman was matched to her own two consecutive births. The main analytic property of this approach is that, by structurally restricting the contrast to within-woman differences, it removes between-mother variation arising from time-invariant maternal-level factors (such as genetic predisposition, anatomic variation, chronic disease background, stable socioeconomic factors, and baseline lifestyle characteristics) without thereby establishing causal identification (see Section 4.6 for the design’s structural limitations). The analysis assessed whether the difference between a woman’s two deliveries was more pronounced among women with short IDI than among those with standard IDI, providing a second, independent inferential axis for evaluating the biological consistency of the IDI association. The design is not a sibling-comparison design in the sense of Ball et al. (2014) [16] or Hanley et al. (2017) [17]—where siblings of different mothers serve as internal controls—but rather a within-mother paired comparison of two consecutive births in the same woman, discussed further in Section 4.6.

Conditional logistic regression was used for binary paired outcomes, with each woman serving as her own matched control. For continuous paired outcomes, the δ value (index birth minus previous birth) was calculated and compared between the short and standard IDI groups using multivariable linear regression. Within-mother models were adjusted only for time-varying variables (e.g., gestational age at the index birth, mode of delivery, induction, and the age difference at the second pregnancy); time-invariant maternal factors were excluded from the adjustment set because they were controlled by design. The consistency, or discordance, between the primary multivariable analyses (Section 2.6.2) and the within-mother sensitivity analyses (Section 2.6.3) is reported transparently and used to identify outcomes for which time-invariant confounding may drive the between-mother signal.

#### 2.6.4. Robustness and Sensitivity Framework

The following subsections describe complementary analytic approaches designed to evaluate the robustness of the primary multivariable inference (Section 2.6.2) against standard epidemiologic assumptions and to triangulate with the within-mother δ-regression sensitivity analysis (Section 2.6.3). These methods are framed as complementary—not competing—within the hierarchy stated in the Section 2.6 preamble; they do not replace the primary inference but provide a supporting sensitivity framework.

#### 2.6.5. Propensity Score Inverse Probability of Treatment Weighting (IPTW)

To relax the parametric assumptions of regression modeling, inverse probability of treatment weighting (IPTW) based on the propensity score was applied as a complementary sensitivity analysis to assess robustness to model specification. The propensity score was estimated from a logistic regression model predicting short IDI status. Stabilized weights were calculated, and extreme weights were truncated at the 1st and 99th percentiles. Weighted outcome models were then fitted using HC1 robust (Huber–White) variance estimation. Covariate balance before and after weighting was assessed using SMDs (Supplementary Table S5). The 6-covariate propensity score model formula, coefficient estimates, and reproduction of the primary IPTW odds ratios are reported in Supplementary Table S11. Full weight diagnostics, the pre- and post-IPTW covariate balance, and the propensity-score distribution and overlap are reported in Supplementary Table S12, which extends Supplementary Table S5.

#### 2.6.6. Small-Cell Correction: Firth Penalized Logistic Regression

Because rare outcomes (e.g., peripartum hysterectomy and neonatal mortality) may be subject to quasi-separation, Firth penalized logistic regression was performed as an additional robustness analysis. This approach reduces small-sample bias in maximum-likelihood estimation under rare-event conditions and yields finite estimates.

#### 2.6.7. Mediation Analysis: Postpartum Anemia as Intermediate

To examine the pathway proposed by the maternal depletion hypothesis—short IDI → postpartum anemia (24 h, Hb < 10 g/dL) → EPDS ≥ 13—a complementary mediation analysis was conducted using the difference method of VanderWeele and Vansteelandt (2010) [18]. The total effect (ORtotal), direct effect (ORdirect), indirect effect (ORindirect = α × β), and proportion mediated [(log ORtotal − log ORdirect)/log ORtotal] were calculated on the log-OR scale. A 95% CI for the proportion mediated was obtained using 10,000 bootstrap resamples with the bias-corrected and accelerated (BCa) method [19] (Supplementary Table S6).

#### 2.6.8. Restricted Cubic Splines: Continuous Dose–Response

To characterize potential nonlinear associations between IDI and the primary outcomes, restricted cubic splines were fitted for IDI in months using 4 degrees of freedom, with knots at the 5th, 35th, 65th, and 95th percentiles and a reference value of 24 months. A test of nonlinearity based on the joint significance of the spline terms was reported.

Sensitivity of the spline estimates to alternative knot configurations (3-, 4-, and 5-knot specifications with varying placement) is reported in Supplementary Table S13; the primary 4-df, 5/35/65/95-percentile specification yielded qualitatively concordant results with the 3-knot and alternative 4-knot specifications. Diagnostic plots for the 5-knot specification (log-odds curves, Pearson residuals vs. fitted values, and IDI distribution by outcome) are provided in Supplementary Figure S2.

#### 2.6.9. Additional Sensitivity Analyses

As part of the broader robustness framework, the following additional sensitivity analyses were conducted: (i) alternative IDI cutoffs (6, 12, and 18 months); (ii) complete-case analyses excluding multiple gestations and missing exposure records; (iii) repetition of the primary analyses on the MICE-imputed dataset; and (iv) calculation of E-values for each primary outcome using the VanderWeele and Ding (2017) formula [20] to assess sensitivity to unmeasured confounding (Supplementary Table S8). Because the study window spans approximately three calendar years, subgroup analyses at IDI thresholds >36 months were not feasible in this cohort and were not conducted.

#### 2.6.10. Multiple Testing Correction

Primary inference was based on the raw *p* value and 95% CI for each outcome, with interpretation emphasizing effect size and uncertainty. As a complementary robustness measure for same-family error control, the Benjamini–Hochberg false discovery rate (BH-FDR) procedure was applied across the 13 pre-specified outcomes [21], and FDR-adjusted q values are presented in Supplementary Table S7.

#### 2.6.11. Sample Size Considerations

An a priori sample size estimation was performed at the time of ethics application using GPower 3.1 for a two-tailed z-test of the difference between two independent proportions. Assumptions were derived from institutional pilot data and the existing literature on short interdelivery intervals and neonatal acid–base outcomes: an expected prevalence of the primary neonatal outcome (umbilical artery pH < 7.10) of 10% in the short IDI arm (p1 = 0.10) versus 4% in the standard IDI arm (p2 = 0.04), with α = 0.05 and 80% power (1 − β = 0.80). To reflect the expected exposure distribution in a tertiary referral center—where short IDI is the more common presentation—an anticipated exposure-group ratio (the cohort-based analog of an allocation ratio in an observational setting) of N2/N1 = 0.337 (standard IDI: short IDI) was specified. Under these assumptions, the minimum required sample size was 599 short IDI and 202 standard IDI women (total *n* = 801). The analytic cohort exceeded this threshold (*n* = 635 short IDI and *n* = 216 standard IDI; total *n* = 851 after post-extraction eligibility verification), and the observed prevalences of the primary neonatal outcome (9.6% in short IDI and 4.6% in standard IDI) closely matched the a priori assumptions. The observed allocation ratio (0.340) also closely matched the planned 0.337. The study was not formally powered for the key secondary outcomes (postpartum anemia and postpartum depression), which were pre-specified secondary endpoints embedded in the same cohort; the effect sizes observed for these outcomes are interpreted accordingly. No additional post hoc power calculations were performed, as retrospective (post hoc) power is a deterministic function of the observed p-value and provides no information beyond it, in line with established methodological guidance. Figure 2 summarizes the analytical strategy as a hierarchy of inference. Primary inference is drawn from the multivariable regression models (Section 2.6.2). All subsequent analyses serve as sensitivity tiers: Tier 1 (propensity score IPTW) addresses model specification; Tier 2 (within-mother δ-regression and E-values) addresses time-invariant and unmeasured confounding. Multiple-testing correction (BH-FDR) is applied across the 13 pre-specified outcomes, and restricted cubic splines characterize the exposure–response shape descriptively without being used for primary inference.

#### 2.6.12. Software and Analytic Platform

Analyses were performed on a hybrid platform. IBM SPSS Statistics version 29 (IBM Corp., Armonk, NY, USA) was used for descriptive statistics, two-group comparisons, the multivariable logistic and linear regression models in Section 2.6.2, and the within-mother conditional logistic and linear models in Section 2.6.3, which together formed the primary inference engine. Python version 3.10.12 (statsmodels 0.14, scipy 1.11, miceforest 5.7, NumPy 1.25, and pandas 2.1) was used for the robustness framework, including IPTW-weighted estimation (Section 2.6.5), Firth penalized regression (Section 2.6.6), difference-method mediation analysis with 10,000-sample BCa bootstrapping (Section 2.6.7), restricted cubic spline models (Section 2.6.8), MICE imputation with Rubin-rule pooling (Section 2.5.5), and BH-FDR correction (Section 2.6.10). The primary models in Section 2.6.2 were cross-validated across both platforms and agreed to two decimal places. GPower 3.1 was used for the a priori sample size estimation described in Section 2.6.11. All tests were two-sided, and *p* < 0.05 was considered statistically significant.

### 2.7. Ethics

#### 2.7.1. Ethics Committee Approval

The study protocol was approved by the Non-Interventional Clinical Research Ethics Committee of Ankara Etlik City Hospital (AEŞH–BADEK2) on 24 March 2026 (decision number AEŞH-BADEK2-2026-257; chair: Prof. Dr. İrfan Şençan). All study procedures were conducted in accordance with the Declaration of Helsinki (Fortaleza revision, 2013).

#### 2.7.2. Helsinki and CIOMS Framework

This study was conducted in accordance with the World Medical Association Declaration of Helsinki (2013) and the Council for International Organizations of Medical Sciences (CIOMS 2016) ethical guidelines for research involving human subjects. CIOMS 2016 Guideline 10 specifically provided the ethical basis for the waiver of informed consent in retrospective record-based studies.

#### 2.7.3. Informed Consent

This study was based exclusively on secondary analysis of electronic health records generated as part of routine clinical care and involved no additional participant contact. The Ethics Committee therefore granted a waiver of individual informed consent in line with CIOMS 2016 Guideline 10 and the relevant provisions of the Declaration of Helsinki. At the time of hospital admission, all patients had signed the institutional general informed consent form permitting retrospective use of records for research purposes.

#### 2.7.4. Data Confidentiality and Security

All patient identifiers were coded using an irreversible matching key immediately after data extraction, and the analytic dataset contained no names, Turkish national identification numbers, chart numbers, or other identifiable fields. Data management complied with the Turkish Personal Data Protection Law (Law No. 6698, KVKK) and institutional data governance policies and was conducted in an encrypted environment with access restricted to the registered study team.

#### 2.7.5. Data Sharing

The anonymized dataset supporting the findings of this study is available from the corresponding author upon reasonable request and subject to approval by the institutional ethics committee. Software versions and analytic procedures are described in this Methods section and in the Supplementary Material.

## 3. Results

### 3.1. Cohort Assembly and Baseline Balance

A total of 851 women who delivered their second birth at Ankara Etlik City Hospital between 1 January 2023 and 31 December 2025 were included, yielding 1702 consecutive singleton live births. Based on IDI, 635 women were classified into the short IDI group (<24 months) and 216 into the standard IDI group (≥24 months).

Baseline characteristics are summarized in Table 1. Most obstetric variables were well balanced between the groups, with standardized mean differences (SMDs) below the pre-specified threshold of 0.10: parity (|SMD| = 0.01), gravidity (0.09), previous cesarean delivery (0.04), cesarean delivery rate (0.07), and labor induction (0.07).

Four covariates showed imbalance: maternal age (|SMD| = 0.22), pre-operative hemoglobin (0.51), pre-operative anemia (0.35), and, marginally, gestational age (0.13). These variables were included both in the propensity score model and in the primary multivariable (MV) logistic regression adjustment set.

Differences in neonatal anthropometric variables—birth weight (|SMD| = 0.31), length (0.29), 1 min Apgar (0.30), and 5 min Apgar (0.18)—were not included in the propensity score or multivariable models, as these variables were treated as outcomes rather than covariates.

### 3.2. Primary Outcomes (n = 2, Pre-Specified)

(i) Umbilical artery pH < 7.10 (neonatal acidosis): Neonatal acidosis occurred in 61/635 (9.6%) newborns in the short IDI group and in 10/216 (4.6%) in the standard IDI group; the unadjusted OR was 2.11 (95% CI 1.07–4.13; *p* = 0.022), and the adjusted OR was 2.37 (95% CI 1.17–4.82; *p* = 0.017; BH-FDR q = 0.075). The IPTW sensitivity analysis yielded an OR of 2.25 (95% CI 1.12–4.53; *p* = 0.023). The direction of effect was consistent across the multivariable and IPTW models, although the q value exceeded the 0.05 threshold. UA pH < 7.10 represents a clinical threshold for asphyxia risk, and its higher prevalence in the short IDI group is directionally consistent with maternal-depletion hypotheses. However, because the FDR-adjusted q value (0.075) does not cross the 0.05 multiple-testing threshold, and because this outcome falls within an analytic family in which the composite signal is largely attributable to the same pH events (88% overlap; see Section 3.3), this finding should be interpreted as exploratory and hypothesis-generating. It does not provide confirmatory evidence and requires independent validation in adequately powered cohorts before any clinical inference is drawn.

(ii) Maternal composite morbidity (MCM; blood transfusion and/or ICU admission). MCM occurred in 107/635 (16.9%) women in the short IDI group and in 25/216 (11.6%) in the standard IDI group; the unadjusted OR was 1.53 (95% CI 0.96–2.43; *p* = 0.065), and the IPTW-adjusted OR was 1.35 (95% CI 0.85–2.16; *p* = 0.204). The multivariable adjusted OR for strict MCM was 1.64 (95% CI 0.85–3.16; q = 0.256), which did not cross the FDR-positivity threshold. The MCM signal therefore remained directionally consistent but did not reach statistical significance in this cohort.

### 3.3. Key Secondary Outcomes (n = 2, Pre-Specified)

(iii) Postpartum anemia (Hb < 10 g/dL at 24 h, clinical action threshold): Postpartum anemia occurred in 325/635 (51.2%) women in the short IDI group and in 62/216 (28.7%) in the standard IDI group; the unadjusted OR was 2.59 (95% CI 1.86–3.61; *p* < 0.001), and the adjusted OR was 1.84 (95% CI 1.26–2.68; *p* = 0.002; *q* = 0.022; FDR-positive). The IPTW sensitivity analysis yielded an OR of 1.86 (95% CI 1.29–2.68; *p* < 0.001).

(iv) Postpartum depression (EPDS ≥ 13 at day 10): Postpartum depression occurred in 147/506 (29.1%) women in the short IDI group and in 26/167 (15.6%) in the standard IDI group; the unadjusted OR was 2.19 (95% CI 1.39–3.46; *p* < 0.001), and the adjusted OR was 1.93 (95% CI 1.20–3.10; *p* = 0.007; q = 0.042; FDR-positive). The IPTW sensitivity analysis yielded an OR of 1.78 (95% CI 1.14–2.78; *p* = 0.011). The proportion of missing data was substantially higher for EPDS than for the other outcomes (178/851; 20.9%); MICE × 20 × 10 multiple imputation with Rubin’s rules was therefore applied. Under imputation, the pooled adjusted OR was meaningfully attenuated relative to the complete-case estimate: OR 1.93 → 1.63 (95% CI 1.02–2.62; *p* = 0.043; FMI = 0.285; Barnard–Rubin df = 164.3). The direction of effect was preserved, but a clear attenuation in effect size emerged; because the lower bound of the MICE confidence interval was 1.02, this result was more sensitive to missing-data assumptions than the other primary outcomes and should therefore be interpreted as hypothesis-supporting rather than hypothesis-confirming.

(v) Composite neonatal adverse outcome (UA pH < 7.10, 5 min Apgar < 7, or NICU admission): The composite outcome occurred in 69/635 (10.9%) newborns in the short IDI group and in 13/216 (6.0%) in the standard IDI group; the unadjusted OR was 1.85 (95% CI 1.01–3.39; *p* = 0.044), and the adjusted OR was 2.06 (95% CI 1.09–3.88; *p* = 0.026; q = 0.084); and the IPTW OR was 1.89 (95% CI 1.02–3.48). The structural interpretation of this composite signal is that 61 of the 69 events (88%) in the short IDI group overlapped with UA pH < 7.10 events. This represents a component-driven composite pattern, in which the composite finding is driven almost entirely by the pH component; the composite outcome should therefore be interpreted not as independent primary evidence but as a clinical-action-level restatement of the UA pH < 7.10 signal. Because 88% of the composite events overlap with UA pH < 7.10 events, the composite outcome does not provide independent statistical evidence and should not be interpreted as a separate confirmatory finding. Both the UA pH < 7.10 and the composite estimates are therefore considered exploratory in this cohort; their interpretation is constrained by the FDR-borderline q values (0.075 and 0.084, respectively) and by the component dependence between the two outcomes. This distinction is further addressed in Section 4.2.

### 3.4. Exploratory Outcomes (Remaining Nine Pre-Specified Outcomes)

**Figure 3:**
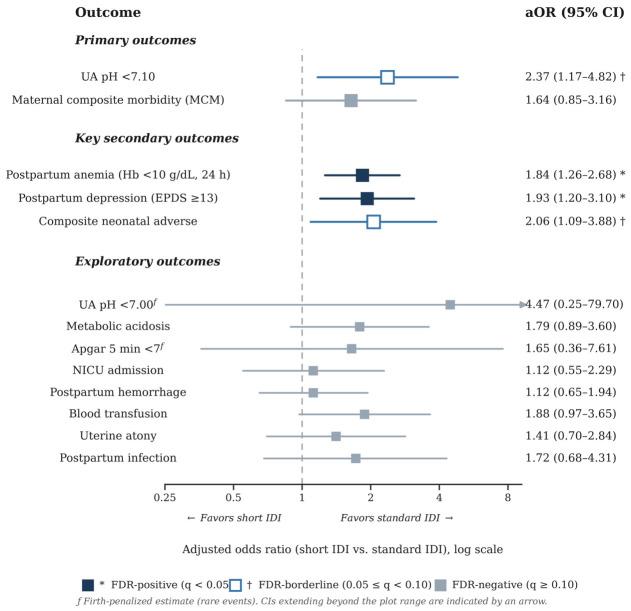
Adjusted odds ratios for the 13 pre-specified outcomes (multivariable logistic regression). Outcomes are grouped according to Table 2: primary outcomes (*n* = 2), key secondary outcomes (*n* = 3), and exploratory outcomes (*n* = 8). Effect estimates are multivariable-adjusted ORs for short IDI (<24 months) vs. standard IDI (≥24 months) with 95% Wald confidence intervals. Color coding indicates FDR status across the 13 outcomes (Benjamini–Hochberg): filled dark squares with * for FDR-positive (q < 0.05); open squares with † for FDR-borderline (0.05 ≤ q < 0.10); gray squares for FDR-negative (q ≥ 0.10). The dashed vertical line marks OR = 1; the x-axis is on a logarithmic scale. Firth penalization (ƒ) is applied when any arm has fewer than five events or the total event count is below 20. For outcomes with very wide confidence intervals (UA pH < 7.00), the CI extending beyond the plot range is indicated by an arrow.

Nine exploratory outcomes were analyzed using the same multivariable model: UA pH < 7.00, metabolic acidosis, 5 min Apgar < 7, and NICU admission (neonatal family); postpartum hemorrhage and blood transfusion (maternal hematologic family); uterine atony and postpartum infection (obstetric family); and maternal ICU admission. None of the exploratory outcomes crossed the FDR threshold of q < 0.05. Because MCM was defined as transfusion and/or ICU admission, blood transfusion is reported separately in Table 2, although the same event may be counted in both the transfusion and MCM columns; this is clarified in the footnote to Table 2 (Figure 3).

### 3.5. Continuous Dose–Response Shape of IDI (Restricted Cubic Spline)

A 4-degree-of-freedom restricted cubic spline analysis modeling IDI as a continuous variable (reference = 24 months; covariates: maternal age, gravidity, parity, gestational age, pre-operative hemoglobin, and pre-operative anemia) is presented in four panels. For UA pH < 7.10 (panel A), the curve rose monotonically below 24 months; at IDI = 18 months the log-OR was approximately 0.5 (OR ≈ 1.6), and at IDI = 12 months it reached approximately 0.7 (OR ≈ 2.0) relative to the 24-month reference (log-OR = 0, OR = 1.0). For postpartum anemia (panel B), the curve was relatively flat across the observed IDI range, with wide confidence bands (log-OR at 12 months ≈ 0.1, OR ≈ 1.1). This pattern is consistent with the non-significant within-mother δ-regression and with the interpretation that a substantial portion of the between-mother anemia signal may reflect time-invariant maternal-level factors (Section 4.3). For EPDS ≥ 13 (panel C), the curve peaked around 12–18 months (log-OR ≈ 0.6–0.7, OR ≈ 1.8–2.0) and then plateaued, suggesting a possible saturation pattern. The composite neonatal outcome (panel D) followed a pattern consistent with UA pH < 7.10 (log-OR at 12 months ≈ 0.7, OR ≈ 2.0). Estimates at the extremes of the IDI distribution (<12 months and >32 months) should be interpreted cautiously because of sparse data in these regions, as reflected in the widening confidence bands. Across all panels, the direction of the curves was consistent around the 24-month threshold.

### 3.6. IPTW Sensitivity Analysis

Re-analysis using stabilized IPTW weights (trimmed at the 1st and 99th percentiles) with HC1-robust standard errors yielded results consistent in direction and magnitude with the primary multivariable model (Table 2, IPTW OR column): postpartum anemia IPTW-OR = 1.86, EPDS IPTW-OR = 1.78, UA pH < 7.10 IPTW-OR = 2.25, and composite neonatal outcome IPTW-OR = 1.89. The primary inferences were therefore robust to the choice of adjustment method (multivariable vs. IPTW).

Model Goodness-of-Fit and Multicollinearity Diagnostics.

Model calibration was evaluated using the Hosmer–Lemeshow goodness-of-fit test (ten risk deciles) for each primary multivariable logistic regression. None of the three primary outcomes showed evidence of lack of fit: postpartum anemia, χ^2^ = 2.79, df = 8, *p* = 0.947 (*n* = 851); EPDS ≥ 13, χ^2^ = 6.32, df = 8, *p* = 0.611 (*n* = 673); UA pH < 7.10, χ^2^ = 13.34, df = 8, *p* = 0.101 (*n* = 851). Variance inflation factors (VIF) for the primary adjustment set were within acceptable bounds for maternal age (1.22), gestational age (1.17), parity (1.20), migrant status (1.06), and induction of labor (1.15). Current cesarean delivery (5.10) and prior cesarean (5.14) approached the conventional threshold (VIF ≥ 5), reflecting the expected shared variance between a history of cesarean and the mode of the index delivery. Sensitivity analyses dropping either variable yielded aOR changes of <5% across the three primary outcomes (Supplementary Table S10); both were therefore retained in the primary model to preserve the pre-specified adjustment set.

### 3.7. Unmeasured Confounding Sensitivity (E-Values)

E-values calculated using the baseline-risk formula of VanderWeele and Ding (2017) [20] were as follows:

- UA pH < 7.10: E-value (point estimate) = 3.88; E-value (CI lower bound) = 1.58.
- Composite neonatal outcome: 3.28; 1.39.
- EPDS ≥ 13: 2.76; 1.60.
- Strict MCM: 2.55; 1.00.
- Postpartum anemia (Hb < 10): 2.32; 1.61.

Point-estimate E-values were in the 2.3–3.9 range, while E-values at the lower 95% CI bounds were in the 1.39–1.61 range. Interpretation in light of plausible unmeasured confounder strengths is provided in Section 4.5.

### 3.8. Mediation Analysis: Postpartum Anemia → EPDS

The pathway of short IDI → postpartum anemia (24 h, Hb < 10 g/dL) → EPDS ≥ 13 was examined using the VanderWeele–Vansteelandt difference method [18]. Because the covariate set for this analysis included mode of delivery, one record with missing delivery mode was excluded (*n* = 672; 10,000 bootstrap samples; BCa intervals):

- Total effect OR = 1.94 (95% BCa CI 1.24–3.23).
- Natural direct effect (NDE) = 1.93 (95% CI 1.25–3.21).
- Natural indirect effect (NIE) = 1.00 (95% CI 0.94–1.07).
- Proportion mediated = 0.6% (95% CI −13.1% to +13.5%).

Within this cohort, the indirect estimate through postpartum anemia is statistically equivalent to zero, and the data therefore do not support maternal hematologic status as the predominant explanatory pathway for the observed IDI–EPDS association. Similar results were obtained for alternative mediators: for ΔHb (pre − 24 h), NIE OR = 0.98 (95% CI 0.90–1.06), proportion mediated −3.2%; for absolute Hb at 24 h, NIE OR = 0.98 (95% CI 0.88–1.05), proportion mediated −3.2%. Possible non-nutritional mechanisms for the observed IDI–EPDS association are addressed in Section 4.4.

### 3.9. Within-Mother Paired δ-Regression Analysis

The paired difference between a woman’s first and second deliveries controls for a substantial portion of time-invariant maternal characteristics (genetic factors, socioeconomic status, baseline family structure). In the paired analysis, postpartum anemia was defined using the same clinical-action threshold (Hb < 10 g/dL at 24 h) applied in the between-mother analysis, allowing direct comparability between the two analyses.

The within-mother paired δ-regression results for all binary and continuous outcomes are summarized in Table 3. Results (paired δ-β coefficients, 95% CIs):

- UA pH < 7.10: β = 0.074 (95% CI 0.026–0.122), *p* = 0.002 (direction preserved).
- Composite neonatal adverse outcome: β = 0.063 (95% CI 0.011–0.116), *p* = 0.019 (direction preserved).
- EPDS ≥ 13 (paired subset, *n* = 516): β = 0.090 (95% CI 0.013–0.167), *p* = 0.021 (direction preserved).
- Postpartum anemia (Hb < 10 g/dL): β = 0.039 (95% CI −0.055 to 0.132), *p* = 0.417 (not significant in the paired analysis).

In the within-mother paired analysis, UA pH < 7.10, the composite neonatal adverse outcome, and EPDS ≥ 13 retained their directional association with short IDI, whereas postpartum anemia did not reach statistical significance. The methodological interpretation of these patterns—including the structural limitations of the within-mother design and the parallel with the sibling-comparison literature—is provided in Section 4.3 and Section 4.6.

### 3.10. Multiple-Testing Correction (BH-FDR)

The Benjamini–Hochberg false-discovery rate (BH-FDR) correction [21] was applied across the 13 pre-specified outcomes:

- FDR-positive (q < 0.05): postpartum anemia (q = 0.022) and EPDS ≥ 13 (q = 0.042).
- Borderline (0.05 ≤ q < 0.10): UA pH < 7.10 (q = 0.075) and composite neonatal adverse outcome (q = 0.084).
- FDR-negative (q ≥ 0.10): all nine remaining outcomes (UA pH < 7.00, q = 0.285; metabolic acidosis, q = 0.241; strict MCM, q = 0.256; blood transfusion, q = 0.256; NICU admission, q = 0.669; postpartum hemorrhage, q = 0.770; uterine atony, q = 0.505; 5 min Apgar < 7, q = 0.505; postpartum infection, q = 0.412).

After correction for multiple testing, the two statistically robust positive findings were postpartum anemia and postpartum depression. The UA pH < 7.10 finding (aOR 2.37, q = 0.075) and the composite neonatal adverse outcome (aOR 2.06, q = 0.084) did not cross the FDR threshold and are therefore reported as exploratory and hypothesis-generating signals requiring independent validation; their interpretation is constrained both by the multiple-testing filter and by the component dependence between the two outcomes (88% overlap; see Section 3.3). A detailed methodological discussion of these exploratory findings is provided in Section 4.2.

### 3.11. Temporal Stability (Enrollment-Quartile Sensitivity)

Because individual-level admission dates were not retained in the de-identified analytic dataset, a strict calendar-quarter sensitivity analysis could not be performed. As a pragmatic proxy, the cohort was split into four equal-sized enrollment quartiles (Q1 earliest to Q4 latest), preserving the case/control enrollment order within each group across the ~3-year study window (January 2023–December 2025). The primary multivariable model was then refit within each quartile for the three primary outcomes (Supplementary Table S9). The direction of association for postpartum anemia remained above 1 across all four quartiles (Q1 aOR 1.91; Q2 3.30; Q3 4.10; Q4 1.76), as did the direction for EPDS ≥ 13 (Q1 3.05; Q2 4.70; Q3 0.91; Q4 2.01) and UA pH < 7.10 (Q1 1.25; Q2 2.21; Q3 2.98; Q4 4.45). Quasi-separation in the sparsest strata—particularly UA pH < 7.10 events in early quartiles—produced non-estimable confidence intervals for five of twelve quartile-specific estimates; this analysis is therefore interpreted as directional rather than inferential. Overall, the primary findings were temporally stable across the enrollment window; a full calendar-date-anchored replication is deferred until re-identified admission dates become available.

## 4. Discussion

### 4.1. Summary of Main Findings

This Discussion section addresses three clinically relevant questions raised in the Introduction. First, whether short interdelivery interval is associated with an objective neonatal acid–base biomarker (umbilical artery pH < 7.10) rather than only with phenotypic endpoints such as preterm birth or low birth weight. Second, whether the maternal evidence base—specifically postpartum hematologic recovery and early depressive symptoms—shows IDI-related signals in a high-income setting. Third, whether any such signals persist after a priori exclusion of high-risk obstetric phenotypes. The findings are interpreted within the multilayered sensitivity framework described in Methods (Section 2.6, Figure 2), with attention to the residual confounding and design-related limitations addressed in Section 4.6.

In this single-center retrospective cohort study with within-mother sensitivity analysis, two consecutive singleton live births from 851 women (1702 records) were analyzed. Women with an interdelivery interval below the World Health Organization (WHO)-recommended 24-month threshold (IDI < 24 months) showed adverse signals of varying magnitude across four a priori outcome domains at their second delivery. After multiple-testing correction (BH-FDR across 13 outcomes), two statistically robust positive findings emerged: postpartum anemia (Hb < 10 g/dL at 24 h; adjusted OR 1.84, 95% CI 1.26–2.68; q = 0.022) and postpartum depression (EPDS ≥ 13 at day 10; adjusted OR 1.93, 95% CI 1.20–3.10; q = 0.042).

Two additional findings showed a directionally consistent pattern but did not cross the FDR threshold and are therefore reported as exploratory: umbilical artery pH < 7.10 (aOR 2.37; q = 0.075), which represents a clinical threshold for asphyxia risk, and the composite adverse neonatal outcome (aOR 2.06; q = 0.084), which is component-driven by the UA pH signal (88% event overlap). These exploratory findings are interpreted in detail in Section 4.2. No FDR-positive signals were identified among the remaining nine exploratory outcomes.

Absolute effect sizes: To contextualize the clinical meaningfulness of these findings beyond relative measures, absolute risk differences (ARDs) and numbers needed to harm (NNH) are reported for the FDR-positive and FDR-borderline outcomes. For postpartum anemia (Hb < 10 g/dL at 24 h), the observed prevalences were 51.2% in the short IDI arm versus 28.7% in the standard IDI arm (ARD +22.5 percentage points; NNH ≈ 4.4). For EPDS ≥ 13 at day 10, the observed prevalences were 29.1% versus 15.6% (ARD +13.5 percentage points; NNH ≈ 7.4). For umbilical artery pH < 7.10 (FDR-borderline), the observed prevalences were 9.6% versus 4.6% (ARD +5.0 percentage points; NNH = 20). These absolute measures indicate that, beyond their relative-risk signals, the postpartum anemia and EPDS associations correspond to clinically substantial frequency differences at the individual short-IDI pregnancy.

The Discussion addresses, in turn, (i) the mechanistic framework for the neonatal acid–base finding and the implications of the overlap between UA pH and the composite outcome; (ii) the discordance of the postpartum anemia finding in the within-mother sensitivity analysis and its parallel with the sibling-comparison literature; (iii) the lack of mediation of the IDI–EPDS association through maternal anemia; (iv) the joint interpretation of the unmeasured-confounding sensitivity, FDR, and MICE findings; (v) the strengths and limitations of the study, including selection bias; and (vi) clinical and public-health implications.

### 4.2. Neonatal Acid–Base Findings—Exploratory Interpretation

The point estimate for UA pH < 7.10 (adjusted OR 2.37) is directionally consistent with the monotonic increase observed in the continuous IDI–pH dose–response curve below 24 months, with the within-mother paired δ-regression (β = 0.074, *p* = 0.002), and with the E-value at the point estimate (3.88). Taken together, these observations indicate a directionally coherent pattern; however, several methodological considerations argue strongly against interpreting this signal as confirmatory.

**Figure 4:**
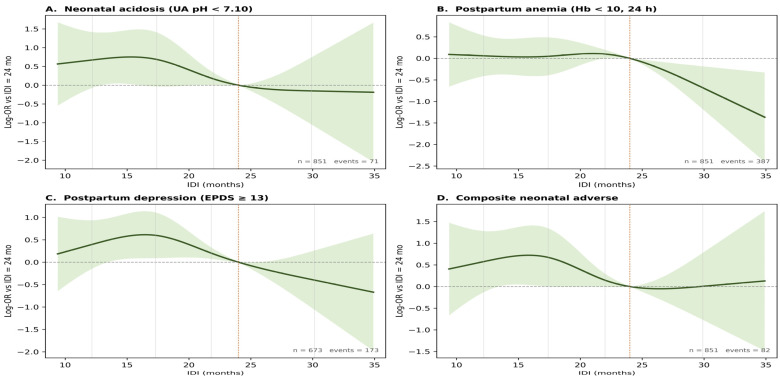
Interdelivery-interval (IDI) dose–response modeled as a restricted cubic spline (4 df), with the reference fixed at 24 months. The four panels show (**A**) umbilical-artery pH < 7.10, (**B**) postpartum anemia (Hb < 10 g/dL at 24 h), (**C**) postpartum depression (EPDS ≥ 13), and (**D**) the composite neonatal adverse outcome. Each curve plots the log-odds-ratio (vs. IDI = 24 months) across the observed IDI range, with 95% Wald confidence bands. The y-axis is on the log-OR scale: log-OR = 0 corresponds to OR = 1.0 (no difference from the 24-month reference); values above zero indicate increased risk at that IDI (e.g., log-OR = 0.7 ≈ OR 2.0; log-OR = 0.5 ≈ OR 1.6); values below zero indicate decreased risk. Light vertical lines mark the knot positions (5th, 35th, 65th, and 95th percentiles of the observed IDI distribution); the dotted vertical line marks the pre-specified 24-month reference. Models are adjusted for maternal age, gravidity, parity, gestational age at index, pre-operative hemoglobin, and pre-operative anemia. Estimates at the extremes of the observed IDI range should be interpreted with caution because of sparse observations, as reflected in the widening confidence bands. In each panel, the dashed horizontal line indicates the null value (odds ratio = 1); the interdelivery interval of 24 months serves as the reference.

First, the FDR-adjusted q value (0.075) does not cross the conventional 0.05 multiple-testing threshold. Although the E-value at the point estimate is moderate, the E-value at the lower confidence bound (1.58) is modest, indicating that an unmeasured confounder of relatively limited strength could shift the interval estimate towards the null. Second, the composite neonatal outcome (aOR 2.06; q = 0.084) is component-driven, with 88% of events overlapping with the UA pH < 7.10 events, and therefore does not provide independent corroborating evidence. Third, the absolute event count in the standard-IDI arm is small (10 events of UA pH < 7.10), which contributes to a wide confidence interval (1.17–4.82) and to estimation instability evident in the spline panel at the upper IDI range (Figure 4).

Accordingly, we interpret the UA pH < 7.10 finding—and, by extension, the component-dependent composite neonatal outcome—as exploratory and hypothesis-generating rather than confirmatory. The directional consistency across the within-mother and dose–response analyses motivates further investigation, but the clinical implications of these findings cannot be drawn from the current cohort. Confirmation will require independent, adequately powered multicenter cohorts in which the neonatal acid–base outcome is pre-specified as a primary endpoint and analytic power is calibrated to a more conservative effect size.

Mechanistically, the association between short IDI and neonatal acid–base disturbance is compatible with two main hypotheses (compatibility rather than proof). The first is the maternal nutritional depletion hypothesis proposed by King (2003) [14], in which insufficient time between pregnancies may prevent adequate replenishment of folate, iron, vitamin B12, and other micronutrient stores. Reduced iron reserves, in particular, may compromise fetal oxygenation by affecting both maternal oxygen-carrying capacity and placental ferritin/hepcidin dynamics. The second is the uteroplacental structural insufficiency hypothesis, in which inadequate endometrial and myometrial remodeling between pregnancies predisposes to placental dysfunction. Both mechanisms point toward chronic subclinical depletion of placental reserve rather than acute intrapartum asphyxia, potentially rendering UA pH < 7.10 a particularly sensitive endpoint.

This mechanistic framework is presented as a hypothesis; direct biochemical or histopathological evidence was not collected in this cohort, and confirmation would require prospective biomarker-based studies.

The composite adverse neonatal outcome (UA pH < 7.10, 5 min Apgar < 7, or NICU admission) showed a concordant signal (aOR 2.06). However, because 88% of the composite events overlap with UA pH < 7.10 events, the composite does not provide statistically independent evidence; both outcomes share the same component-driven uncertainty discussed above and should be regarded jointly as exploratory.

### 4.3. Maternal Anemia Findings, Within-Mother Discordance, and the Sibling-Comparison Literature

Postpartum anemia was the strongest maternal signal remaining robust after multiple-testing correction (aOR 1.84; q = 0.022; IPTW-OR 1.86; prevalence 51.2% vs. 28.7%). This finding aligns with the broader literature: maternal iron-depletion models [1,22] offer a biologically plausible explanation linking short IDI to inadequate restoration of iron stores between pregnancies, and the pattern is consistent with the framework of Milman (2011) [23], in which repeated short-interval pregnancies progressively erode maternal iron reserves.

In contrast, the within-mother δ-regression yielded a non-significant estimate for postpartum anemia (β = 0.039, 95% CI −0.055 to 0.132; *p* = 0.417). Two factors may account for this discrepancy, each warranting cautious interpretation: (a) the within-mother design has reduced statistical power, particularly in a short-IDI-dominant cohort with a limited standard IDI reference group (*n* = 216); and (b) because the within-mother design controls for time-invariant maternal characteristics (genetic variation in iron metabolism, baseline nutritional status, dietary patterns), the possibility that part of the between-mother estimate reflects these time-invariant factors cannot be excluded.

Parallel with the sibling-comparison literature: This discordance is directly parallel to the insight articulated by Ball et al. (2014) [16], Hanley et al. (2017) [17], and Class et al. (2017) [24] using sibling-comparison designs: between-mother associations for short interpregnancy and interdelivery intervals are substantially attenuated—and in several outcome domains rendered null—once time-invariant maternal-level confounding is removed by within-family comparisons. The parallel in our cohort is most pronounced for postpartum anemia, where time-invariant maternal hematologic biology (e.g., baseline iron metabolism, dietary patterns, chronic inflammation) is a strong candidate source of residual between-mother confounding. The within-mother δ-regression, however, retained directional consistency—and, for UA pH < 7.10, statistical significance (β = 0.074, *p* = 0.002)—for the neonatal acid–base outcome, diverging from the typical sibling-comparison attenuation pattern. This suggests that the neonatal acid–base signal is less likely to be driven by time-invariant maternal confounding than the maternal hematologic signal. The design used here is not a sibling-comparison design as understood in Ball/Hanley/Class—where siblings of different mothers serve as internal controls within a family-fixed-effects framework—but rather a within-mother paired comparison of two consecutive births in the same woman; the interpretive logic, however, is the same.

The maternal hematologic findings should therefore be interpreted with caution: the between-mother estimate is a clinically meaningful and statistically robust signal, but it may partly reflect unmeasured time-invariant maternal heterogeneity. The within-mother analysis is regarded as a complementary sensitivity analysis within the analytic hierarchy (Section 2.6 preamble) rather than as a confirmatory analysis; the scope and structural limitations of this design are discussed in detail in Section 4.6.

Pre-operative hemoglobin as an effect modifier and mediator: When pre-operative hemoglobin was added as a continuous covariate to the primary multivariable model for 24 h anemia, each 1 g/dL higher pre-op Hb was associated with markedly lower odds of postpartum anemia (aOR 0.24, 95% CI 0.19–0.31; *p* < 0.001; *n* = 851). On a per-standard-deviation basis (0.74 g/dL), this corresponds to an aOR of 0.35 (95% CI 0.29–0.42). The magnitude of this association—substantially larger than the short-IDI effect itself (aOR 1.84)—is consistent with a mediation structure in which inadequate interpregnancy iron recovery is the proximate driver of the 24 h anemia phenotype, while short IDI operates primarily through its downward pressure on pre-op Hb. This finding motivated the formal mediation analysis reported in Supplementary Table S6. The clinical actionability of this pathway is supported by the Cochrane systematic review of Peña-Rosas et al. (2015) [25], which pooled 61 randomized trials (44 trials with 43,274 women) and demonstrated that daily oral iron supplementation during pregnancy reduces maternal anemia at term by 70% (pooled RR 0.30, 95% CI 0.19–0.46) and iron deficiency by 57% (RR 0.43, 0.27–0.66)—providing the evidentiary basis for the targeted iron supplementation strategies advanced in Section 4.7.

### 4.4. Postpartum Depression and Non-Hematologic Pathways

The EPDS ≥ 13 finding (aOR 1.93; q = 0.042) remained statistically robust after multiple-testing correction. After MICE × 20 multiple imputation, however, the pooled estimate was attenuated to OR 1.63 (95% CI 1.02–2.62; *p* = 0.043). Because the lower bound of the confidence interval fell to 1.02, this finding is more sensitive to missing-data assumptions than the other primary outcomes, and we therefore interpret it as hypothesis-supporting rather than hypothesis-confirming.

The mediation analysis using the VanderWeele–Vansteelandt difference method [18] further characterized this association: along the hypothesized short IDI → postpartum anemia → EPDS pathway, the indirect OR was 1.00 (95% CI 0.94–1.07), with a proportion mediated of 0.6% (95% CI −13.1% to +13.5%). Similar results were observed for alternative mediators (ΔHb and absolute Hb at 24 h; NIE ≈ 0.98 in both). These findings do not support, within this cohort, the hypothesis that postpartum iron depletion is the dominant mechanistic pathway linking short IDI to postpartum depression.

Rather than excluding this mechanism entirely, it is more appropriate to conclude that its role as a primary explanatory pathway appears unlikely in this cohort. Alternative non-nutritional mechanisms may better explain the observed association: (i) sleep disruption and chronic sleep deprivation—women with short IDI may still be engaged in breastfeeding and nighttime care when a second newborn arrives, potentially affecting prefrontal–amygdala balance and hypothalamic–pituitary–adrenal (HPA) axis function [26]; (ii) allostatic load and neuroendocrine depletion—repeated cycles of pregnancy, delivery, and lactation may produce cumulative alterations in cortisol, prolactin, and estrogen dynamics [27]; (iii) psychosocial stress and caregiving burden—simultaneously caring for two infants, economic strain, and limited social support are independent risk factors [28]; and (iv) reproductive hormonal sensitivity—genetic or epigenetic susceptibility to postpartum estrogen withdrawal in some women [29].

From a clinical perspective, this distinction is relevant for management priorities: the association between short IDI and postpartum depression in this cohort does not appear to be substantially mediated by maternal hematologic status, and iron supplementation alone is therefore unlikely to address it. Early psychosocial screening, sleep-support interventions, and family-centered strategies to redistribute the caregiving burden represent more plausible and targeted approaches. The clinical importance of identifying postpartum depression in this context is underscored by the meta-analytic evidence of Grote et al. (2010) [30], which pooled data from 29 prospective studies and demonstrated that categorically defined antenatal depression is associated with significant increases in preterm birth (pooled RR 1.39, 95% CI 1.19–1.61), low birth weight (RR 1.49, 1.25–1.77), and intrauterine growth restriction (RR 1.45, 1.05–2.02)—supporting the case for universal screening and early intervention in high-risk pregnancies, including those with short IDI.

### 4.5. Methodological Sensitivity: E-Values, FDR, and MICE—Limits of Interpretation

This study was conducted within a multilayered sensitivity framework that explicitly acknowledges the limitations of an observational design: multivariable adjustment (primary) + IPTW (method independence) + E-values (robustness to unmeasured confounding) + Firth penalization (rare events) + MICE × 20 (missing data) + BH-FDR (multiple testing) + paired δ-regression (time-invariant confounding). This architecture protects against reliance on any single methodological assumption.

Even so, this layered approach cannot exclude residual confounding. E-value analysis indicated that the point estimates (range 2.3–3.9) would require an unmeasured confounder stronger than the most prominent observed association in this cohort (pre-operative anemia → postpartum anemia, RR ≈ 3.2) to fully explain the findings, suggesting reasonable robustness at the level of the point estimates. By comparison, E-values at the lower confidence-interval bounds dropped to 1.39–1.61, a range that overlaps with theoretically plausible confounder strengths. Accordingly, we interpret the primary findings as moderately robust at the level of the point estimates but vulnerable at the limits of the confidence intervals.

BH-FDR across all 13 outcomes is a more liberal alternative to Bonferroni correction; it controls the false discovery rate while still addressing concerns about multiplicity. For UA pH < 7.10, a q value of 0.075 transparently indicates that the finding did not cross the conventional multiple-testing threshold and is therefore reported as exploratory and hypothesis-generating (see Section 4.2 for the detailed exploratory interpretation).

MICE analysis introduced a substantive adjustment only for EPDS (the other primary outcomes were fully or nearly fully observed). The attenuation in effect size for EPDS (OR 1.93 → 1.63) suggests that the missingness may not be completely random and could be partly outcome-related, which further emphasizes the need for independent replication of this finding.

EPDS missingness structure and the missing-at-random assumption: EPDS was unavailable at the index delivery for *n* = 178/851 (20.9%) women. The MICE × 20 multiple imputation reported in Section 2.6.5 and Supplementary Table S4 relies on the missing-at-random (MAR) assumption—that, conditional on the observed covariates, the probability of missingness is independent of the unobserved EPDS value. Because this assumption is not directly testable, we evaluated its plausibility in two ways.

First, a comparison of demographic and obstetric characteristics between EPDS-available (*n* = 673) and EPDS-missing (*n* = 178) women is reported in Supplementary Table S3b. Migrant status differed materially between the two groups (SMD = −0.22; *p* = 0.009), with non-Turkish-citizen women more likely to be missing EPDS data at day 10—consistent with language, transport, and continuity-of-care barriers in postpartum follow-up. No other variable differed at |SMD| > 0.15. Because migrant status was both predictive of missingness and included as a covariate in the MICE imputation model, the MAR assumption is partly defensible: conditional on observed migrant status (together with maternal age, gestational age, parity, mode of delivery, and pre-operative anemia, all of which were included in the imputation model), the residual MAR violation should be reduced. However, MAR cannot be fully verified, and an unmeasured component of the missingness mechanism likely remains.

Second, the attenuation of the EPDS point estimate under MICE (OR 1.93 → 1.63; lower 95% CI bound dropping to 1.02) is directionally consistent with missingness that is partly outcome-related (missing-not-at-random, MNAR) rather than strictly MAR—that is, women with unobserved high EPDS scores may have been more likely to miss the postpartum day-10 visit, biasing the complete-case estimate upward and the MICE-imputed estimate toward (but not all the way to) the true value. Under such a MNAR mechanism, the true population EPDS effect is most plausibly bounded between the complete-case estimate (OR 1.93, upper plausibility bound) and a value below the MICE-imputed estimate (OR 1.63, conservative bound). Because the MICE confidence interval’s lower bound (1.02) approaches the null, the EPDS finding is sensitive to the MAR assumption and is therefore interpreted as hypothesis-supporting rather than hypothesis-confirming. A formal sensitivity analysis under specific MNAR patterns (e.g., pattern-mixture or delta-adjustment models) is identified as an a priori future analysis once external EPDS validation data become available.

The within-mother δ-regression for EPDS uses a still smaller subset (*n* = 516) that requires EPDS to be available for both the index and previous delivery; this further sub-cohort structure is annotated in Figure 1, and the corresponding selection constraints are discussed in Section 4.6 (within-mother design—scope and structural limitations).

### 4.6. Strengths and Limitations

The strengths of the study include the following: (i) a cohort design with within-mother sensitivity analysis comparing two consecutive deliveries within the same woman at a single center; (ii) BH-FDR correction across 13 pre-specified outcomes from four outcome domains (neonatal acid–base status, maternal composite morbidity, postpartum anemia, and postpartum depression); (iii) robustness to adjustment strategy through a dual multivariable + IPTW approach; (iv) Firth penalization for rare events; (v) MICE × 20 multiple imputation for missing EPDS data; (vi) E-value analysis for unmeasured confounding; (vii) mechanistic coherence supported by maternal–fetal parallel findings for neonatal acidosis; (viii) modeling of IDI both categorically and continuously (4-df RCS); and (ix) transparent reporting of structural inconsistencies in outcome interpretation (component-driven composite, and paired vs. between-mother differences).

Limitations include the following: (a) the single-center design limits external validity—findings from a tertiary hospital in Türkiye require validation in European and North American cohorts; (b) the retrospective observational design precludes causal inference, and the associations should therefore be interpreted as “short IDI is associated with adverse outcomes” rather than as causal; (c) the 24-month threshold aligns with the WHO recommendation but is not data-driven, which is why continuous RCS analysis is provided as a complementary approach; (d) the lack of formal preregistration, addressed by detailed ex ante protocol documentation in Section 2; (e) EPDS was assessed only on day 10, limiting inference for longer-term postpartum depression phenotypes; (f) data processing followed a hybrid SPSS + Python workflow, with raw outputs and syntax documented in Section 2.6; and (g) the absence of key covariates such as paternal characteristics, detailed socioeconomic variables, and duration of breastfeeding, which may represent sources of residual confounding.

Generalizability—single-center design and study period: The findings of this study are derived from a single tertiary public hospital in Ankara, Türkiye, over a narrow study window (January 2023 to December 2025). Three structural features should temper the geographic and temporal generalizability of the estimates. First, the institutional case mix reflects a public-tertiary referral pattern in the Turkish health system; the prevalence of short IDI in this cohort (74.6%) is high relative to most European and North American settings, plausibly reflecting both local family-planning and fertility patterns and the migrant subgroup composition (30.0% non-Turkish nationality). The absolute prevalences of postpartum anemia (51.2% vs. 28.7%), EPDS ≥ 13 (29.1% vs. 15.6%), and UA pH < 7.10 (9.6% vs. 4.6%) should therefore not be directly extrapolated to populations with substantially different baseline risk profiles. Second, although the within-cohort effect estimates (adjusted ORs) may be more transportable than the absolute prevalences, the residual confounding and selection-bias considerations discussed above apply to the same cohort and may operate differently in other settings. Third, the 36-month study window does not permit examination of longer-term temporal trends, and the within-quartile sensitivity analysis (Section 3.11, Supplementary Table S9) provides only directional rather than formal temporal stability evidence. Independent replication in geographically diverse, multicenter cohorts—particularly in high-income settings with lower baseline short-IDI prevalence and in other low- and middle-income contexts—is therefore essential to assess the external validity of these findings.

Selection bias—same-center index and previous delivery: Only women for whom both the previous and the index delivery occurred at Ankara Etlik City Hospital were eligible. This design decision ensured within-institution comparability of delivery documentation and laboratory assays, but, by design, this requirement excluded mobile and migrant populations whose first delivery occurred at another institution. Despite this restriction, the retained cohort still includes a substantial migrant subgroup: *n* = 255/851 women (30.0%) were registered with non-Turkish nationality—predominantly Syrian (171/255) and Iraqi (61/255). The migrant proportion was nominally higher in the short IDI group (198/635, 31.2%) than in the standard IDI group (57/216, 26.4%). Migrant status was included as a covariate in every primary multivariable model. The direction of residual selection bias from women whose first delivery was elsewhere is not determinate a priori and depends on the assumed correlation between mobility and both exposure and outcome. If the excluded women tend to have shorter IDI and worse outcomes (e.g., under socioeconomic stress driving both mobility and adverse perinatal signals), their exclusion would attenuate the observed associations, rendering the present estimates conservative (bias toward the null). If the excluded women are on average lower-risk (e.g., healthier migrant workers returning to other institutions), their exclusion would produce bias in the opposite direction. A definitive assessment requires individual-level data on the excluded population, which was not available to the present study. Multicenter replication that explicitly captures the mobile/migrant subset is therefore warranted. In addition to the same-institution eligibility requirement, the within-mother sub-cohort imposes the further constraint that paired outcome data must be available for both deliveries; the structural implications of this constraint are discussed in the “Within-mother design—scope and structural limitations” section above.

Residual confounding: Despite a multilayered adjustment strategy (multivariable adjustment, IPTW, within-mother δ-regression, E-values), several important confounders could not be included in the adjustment set and represent the dominant source of residual bias in this cohort. The DAG (Supplementary Figure S1) identifies four such variables as dashed unmeasured nodes: maternal body-mass index (BMI), smoking, socioeconomic status (SES), and breastfeeding duration. Their plausible directional effects on the observed estimates are summarized below.

Smoking is associated with both shorter IDI (through reduced fertility-management adherence and socioeconomic gradients) and with adverse neonatal acid–base outcomes (through placental vasoconstriction and chronic fetal hypoxia); uncontrolled smoking would therefore tend to inflate the neonatal-acidosis estimate, biasing the IDI–UA pH association away from the null. Body-mass index has a less determinate direction: higher BMI is associated with reduced fertility (longer IDI) but also with postpartum anemia, gestational and intrapartum complications, and depressive symptoms; the net direction of residual confounding by BMI is therefore ambiguous and outcome-dependent. Socioeconomic status is associated with both shorter IDI (through limited access to contraception) and higher EPDS scores (through psychosocial stress, caregiving burden, and reduced access to support); uncontrolled SES is therefore a plausible confounder of the IDI–EPDS association, with the likely direction of bias being away from the null. Breastfeeding duration is associated with longer postpartum amenorrhea (and thus longer IDI) and with maternal iron and emotional status; its omission may contribute residual confounding for both the postpartum anemia and EPDS outcomes, with an indeterminate net direction.

The E-value sensitivity analysis (Section 2.6.9; Supplementary Table S8) provides an explicit quantification of the strength such unmeasured confounders would require. Point-estimate E-values (2.32–3.88) exceed the strongest observed in-cohort association, suggesting that no single unmeasured confounder of the magnitude commonly observed in obstetric cohorts could fully nullify the primary findings. However, E-values at the lower 95% confidence-interval bounds fall to 1.39–1.61, a range that overlaps with plausible confounder strengths for BMI, smoking, and SES, particularly in the EPDS and UA pH < 7.10 analyses. Accordingly, the primary findings should be interpreted as moderately robust at the level of the point estimates but vulnerable to residual confounding at the limits of the confidence intervals, and the associations described in this study are not interpreted as causal effects.

Within-mother design—scope and structural limitations: The within-mother δ-regression, by design, controls for the full set of time-invariant maternal characteristics (genetic background, anatomic and constitutional traits, baseline socioeconomic position, stable dietary patterns, long-term lifestyle factors). This is the principal interpretive strength of the design and the basis for its inclusion as a sensitivity tier in the analytic hierarchy (Figure 2; Section 2.6). However, three structural limitations should be acknowledged when interpreting the within-mother estimates.

(i) Time-varying confounders are not addressed by this design. Between two consecutive deliveries, several maternal characteristics may change in ways that are themselves correlated with both interdelivery interval and outcome: maternal age and parity advance by definition; pre-pregnancy body-mass index may increase or decrease; smoking status, contraceptive method, and breastfeeding duration after the first delivery may all shift; chronic conditions (hypothyroidism, mood disorders, gestational diabetes that became overt) may emerge or be newly diagnosed; and the family’s socioeconomic position, housing, and household composition may evolve. Because the within-mother contrast removes only time-invariant heterogeneity, residual confounding from these time-varying factors persists. The within-mother estimates should therefore not be interpreted as fully causal even where they remain directionally consistent with the between-mother analyses.

(ii) Intercurrent maternal events between the two pregnancies can introduce paired-design bias. Events occurring in the interpregnancy interval—postpartum hemorrhage at the first delivery, subsequent iron repletion or its failure, postpartum depression resolved or persisting, breastfeeding cessation, infection during the puerperium, pregnancy loss preceding the index delivery—were not systematically captured in this dataset. Such intercurrent events may differentially affect the index-delivery outcome in a manner correlated with the duration of the interdelivery interval (e.g., a short interval combined with unresolved postpartum anemia from the first delivery may amplify the apparent IDI–anemia association). The within-mother analysis cannot adjust for these unmeasured between-pregnancy changes.

(iii) The paired design imposes selection constraints that may differ from the cohort as a whole. Women contributing to the within-mother analysis must have completed two consecutive pregnancies at the same institution within the study window and have non-missing data for the paired outcome at both deliveries. For EPDS, this requirement reduces the analytic sample to *n* = 516, and selection is necessarily conditional on attendance at both postpartum day-10 visits—a behavior that may itself correlate with depression risk and with social support. The within-mother estimates therefore apply to a constrained subset of women rather than to the full analytic cohort, and the magnitude of selection effects in this subset cannot be quantified from within-cohort data.

Taken together, these three limitations imply that the within-mother δ-regression should be interpreted as a sensitivity analysis aimed at removing time-invariant maternal confounding, not as a comprehensive causal-effect estimator. Convergence between the within-mother and between-mother estimates strengthens the directional inference (as observed for UA pH < 7.10), while divergence flags time-invariant maternal heterogeneity as a candidate explanation but does not by itself establish such heterogeneity (as observed for postpartum anemia). The interpretation of all within-mother estimates in this manuscript is calibrated to this scope.

Composite outcome definition: The composite adverse neonatal outcome is defined as a three-component composite (UA pH < 7.10, 5 min Apgar < 7, NICU admission); “metabolic acidosis” is presented as a separate exploratory outcome and is not a component of the composite. This distinction is made explicit in Section 2.4.1 and in Supplementary Table S2.

Causal-language disclaimer: The combined use of inverse probability of treatment weighting and within-mother δ-regression in this study may imply, in part, a causal-effect estimation intent. The intent of this multilayered design, however, is to triangulate evidence across methodologies that rely on different identifying assumptions—not to assert causal identification. Given the persistence of plausible residual confounding (Section 4.6) and the FDR-borderline status of the neonatal acid–base outcomes (Section 3.2 and Section 4.2), the effect estimates presented throughout this manuscript should be read as adjusted associations, not as causal effects. Causal inference would require either an experimental intervention (e.g., randomized trials of postpartum contraceptive interventions, Section 4.7) or a quasi-experimental design with a credible source of exogenous variation in birth spacing.

### 4.7. Clinical and Public-Health Implications and Future Research Agenda

The practical implications of this study can be considered at three levels.

At the clinical level, for second deliveries with an IDI < 24 months, the FDR-robust signals support the following considerations: (a) routine 24 h hemoglobin assessment may be standardized, with proactive iron supplementation considered at Hb < 10 g/dL; and (b) day-10 EPDS screening may be paired with targeted psychosocial interventions focused on sleep support and redistribution of the caregiving burden. The neonatal acid–base finding (UA pH < 7.10) did not cross the multiple-testing threshold and is not advanced as a basis for changes in intrapartum monitoring; its exploratory nature precludes clinical recommendations pending confirmatory data. Importantly, treating maternal anemia alone is unlikely to substantially reduce postpartum depression risk (proportion mediated 0.6%); these outcomes should therefore be managed along distinct clinical pathways.

At the public-health level, the WHO recommendation of a 24-month birth interval is supported by these findings; however, this recommendation should be operationalized not through individual blame but through improved access to family planning, effective postpartum contraceptive counseling, and system-level postpartum support. A substantial share of short IDI cases reflects limited access to contraception rather than individual choice, particularly in socioeconomically disadvantaged populations. These findings should therefore be interpreted as a rationale for strengthening system-level postpartum contraceptive services rather than for labeling individuals.

Future research priorities include the following: (1) replication of these findings in multicenter prospective cohorts across diverse geographic settings, particularly for EPDS given its attenuation after MICE; (2) mechanistic studies integrating placental histopathology and iron/hypoxia biomarkers to clarify the UA pH < 7.10 pathway; (3) mediator-focused studies directly measuring sleep, allostatic load, and caregiving burden in the IDI–EPDS relationship; and (4) randomized trials of postpartum contraceptive interventions, which represent one of the most rigorous approaches to testing the causal pathway linking short IDI to adverse outcomes.

Clinical bottom line: For a second pregnancy with an interdelivery interval below the WHO 24-month threshold in our cohort, the absolute risk of postpartum anemia (Hb <10 g/dL at 24 h) was 51.2% versus 28.7% (number needed to harm [NNH] ≈ 4.4), and the absolute risk of postnatal depression (EPDS ≥ 13 at day 10) was 29.1% versus 15.6% (NNH ≈ 7.4). For umbilical artery pH < 7.10, an exploratory finding that did not cross the FDR threshold, the absolute risk was 9.6% versus 4.6% (NNH = 20) and is not advanced as a basis for changes in intrapartum management. Day-10 EPDS screening and 24 h hemoglobin assessment, paired with targeted iron repletion and sleep- and caregiving-support interventions, are the clinically actionable outputs of these findings; the IDI–depression association does not appear to be mediated by maternal hematologic status (proportion mediated 0.6%), so these two outcomes warrant distinct management pathways.

## 5. Conclusions

In this single-center retrospective cohort with within-mother sensitivity analysis, an interdelivery interval shorter than 24 months was associated with two statistically robust outcomes after multiple-testing correction (postpartum anemia and postpartum depression). The neonatal acid–base findings (UA pH < 7.10 and the component-dependent composite neonatal outcome) did not cross the FDR threshold and are therefore reported as exploratory and hypothesis-generating; their interpretation requires confirmation in independent cohorts. The mediation analysis did not support maternal hematologic pathways as the dominant mechanism linking IDI to EPDS; future mechanistic research is therefore directed toward sleep, allostatic-load, and caregiving-burden pathways.

The findings are moderately robust at the level of the point estimates but remain vulnerable to residual confounding at the limits of the confidence intervals; independent multicenter validation and randomized trials of postpartum contraceptive interventions are therefore warranted. Clinically, these results should not be read as assigning responsibility to individual women but as supporting system-level strengthening of postpartum family-planning services and risk-informed postpartum care.

## Figures and Tables

**Table 1:** Baseline maternal and obstetric characteristics at the index (second) delivery, stratified by interdelivery interval (IDI <24 months vs. ≥24 months). Standardized mean differences (SMD, absolute value) are presented; |SMD| > 0.10 indicates clinically relevant imbalance. The IDI row shows “N/A (exposure)” because the variable defines the strata. Variables labeled “MATERNAL” reflect pre-gravid or peripartum maternal status; variables labeled “NEONATAL” reflect outcomes of the index delivery. *p*-values are from Student’s *t*-test or Mann–Whitney U test for continuous variables and χ^2^ or Fisher’s exact test for categorical variables.

Characteristic	Short IDI (<24 mo) *n* = 635	Standard IDI (≥24 mo) *n* = 216	*p*-Value	SMD
**MATERNAL**				
Maternal age (years), mean (SD)	25.9 (4.7)	26.9 (4.9)	0.006	−0.218
Gravidity, mean (SD)	3.0 (1.2)	2.9 (1.1)	0.224	0.094
Parity, mean (SD)	2.7 (1.0)	2.7 (1.0)	0.861	0.014
IDI (months), mean (SD)	17.6 (3.6)	27.6 (2.7)	<0.001	N/A (exposure)
Migrant status, *n* (%)	198 (31.2%)	57 (26.4%)	0.189	0.106
**DELIVERY**				
Gestational age at delivery (weeks), mean (SD)	38.6 (1.3)	38.7 (1.2)	0.088	−0.131
Cesarean delivery, *n* (%)	250 (39.4%)	93 (43.1%)	0.391	−0.074
Induction of labor, *n* (%)	106 (16.7%)	31 (14.4%)	0.483	0.065
Prior cesarean, *n* (%)	222 (35.0%)	79 (36.9%)	0.664	−0.041
**PRE-DELIVERY LABS**				
Hemoglobin, pre-op (g/dL), mean (SD)	11.38 (0.72)	11.76 (0.75)	<0.001	−0.512
WBC, pre-op (×10^9^/L), mean (SD)	9.59 (1.75)	9.42 (1.74)	0.231	0.094
Platelets, pre-op (×10^9^/L), mean (SD)	260 (70)	263 (71)	0.550	−0.047
Pre-operative anemia (Hb < 11 g/dL), *n* (%)	187 (29.4%)	33 (15.3%)	<0.001	0.345
**NEONATAL**				
Birth weight (g), mean (SD)	3216 (367)	3334 (389)	<0.001	−0.312
Birth length (cm), mean (SD)	50.1 (1.9)	50.6 (1.8)	<0.001	−0.292
Apgar 1 min, mean (SD)	7.7 (1.2)	8.0 (1.0)	<0.001	−0.300
Apgar 5 min, mean (SD)	8.9 (0.9)	9.0 (0.8)	0.023	−0.175
Infant sex—Male, *n* (%)	309 (48.7%)	109 (50.5%)	0.664	−0.036
Infant sex—Female, *n* (%)	326 (51.3%)	107 (49.5%)	0.664	0.036

**Table 2:** Between-group comparison of all 13 pre-specified outcomes at the index (second) delivery: short IDI (<24 months) vs. standard IDI (≥24 months). Primary outcomes are shown in the upper block; key secondary and exploratory outcomes in the lower block. Unadjusted, multivariable-adjusted (MV-aOR), and inverse-probability-of-treatment-weighted (IPTW, HC1-robust) odds ratios (ORs) are reported with 95% confidence intervals. BH-FDR q values across the 13 outcomes are summarized in Supplementary Table S7.

Outcome	Short IDI Events/*n* (%)	Standard IDI Events/*n* (%)	Unadjusted OR [95% CI]	MV-aOR [95% CI]	IPTW OR [95% CI]
**PRIMARY OUTCOMES**					
Neonatal acidosis (UA pH < 7.10)	61/635 (9.6%)	10/216 (4.6%)	2.11 [1.07–4.13]	2.37 [1.17–4.82]	2.25 [1.12–4.53]
Maternal composite morbidity (MCM)	107/635 (16.9%)	25/216 (11.6%)	1.53 [0.96–2.43]	1.64 [0.85–3.16]	1.35 [0.85–2.16]
**KEY SECONDARY OUTCOMES**					
Postpartum anemia (Hb < 10 g/dL, 24 h)	325/635 (51.2%)	62/216 (28.7%)	2.59 [1.86–3.61]	1.84 [1.26–2.68]	1.86 [1.29–2.68]
Postpartum depression (EPDS ≥ 13)	147/506 (29.1%)	26/167 (15.6%)	2.19 [1.39–3.46]	1.93 [1.20–3.10]	1.78 [1.14–2.78]
Composite neonatal adverse	69/635 (10.9%)	13/216 (6.0%)	1.85 [1.01–3.39]	2.06 [1.09–3.88]	1.89 [1.02–3.48]
**EXPLORATORY OUTCOMES**					
UA pH < 7.00 ^f^	6/635 (0.9%)	0/216 (0.0%)	4.47 [0.25–79.70] ^f^	NE	5.61 [0.46–68.07] ^f^
Metabolic acidosis	51/635 (8.0%)	10/216 (4.6%)	1.73 [0.88–3.43]	1.79 [0.89–3.60]	1.66 [0.84–3.27]
Apgar 5 min < 7	11/635 (1.7%)	2/216 (0.9%)	1.58 [0.40–6.26]	1.65 [0.36–7.61]	1.72 [0.47–6.33]
NICU admission	34/635 (5.4%)	11/216 (5.1%)	1.02 [0.52–2.04]	1.12 [0.55–2.29]	1.15 [0.56–2.36]
Postpartum hemorrhage	72/635 (11.3%)	19/216 (8.8%)	1.30 [0.77–2.20]	1.12 [0.65–1.94]	1.08 [0.65–1.82]
Blood transfusion	70/635 (11.0%)	12/216 (5.6%)	2.04 [1.09–3.80]	1.88 [0.97–3.65]	1.83 [0.98–3.45]
Uterine atony	47/635 (7.4%)	10/216 (4.6%)	1.59 [0.80–3.16]	1.41 [0.70–2.84]	1.25 [0.65–2.40]
Postpartum infection	29/635 (4.6%)	6/216 (2.8%)	1.58 [0.66–3.74]	1.72 [0.68–4.31]	1.88 [0.72–4.93]

**Footnote.** MCM is defined as blood transfusion and/or maternal ICU admission; because MCM is a compound of transfusion and ICU admission, the transfusion row can also contribute to MCM events, and these should not be interpreted additively. ^f^ indicates Firth-penalized estimate (zero events in one arm or total events < 20); for UA pH < 7.00 the MV-aOR is reported as “NE” (non-estimable) because the logit model did not converge (0 events in the standard-IDI arm), and the unadjusted and IPTW estimates are Firth-penalized with wide confidence intervals and should be interpreted as hypothesis-generating only. UA = umbilical artery; EPDS = Edinburgh Postnatal Depression Scale; NICU = neonatal intensive care unit; NE = non-estimable.

**Table 3:** Within-mother paired δ-regression comparing the index (second) delivery with the same mother’s previous delivery. Binary outcomes were analyzed by conditional (paired) McNemar models and linear Δ-regression on the delivery-level difference; continuous outcomes were analyzed by Δ-regression of the within-mother difference. β coefficients from δ-regression with 95% CIs; models adjusted for time-varying covariates only (gestational age at index, mode of delivery, induction, maternal age at second delivery).

**(a) Binary Outcomes**					
**Outcome**	***n* Pairs (Short|std)**	**Incidence Δ Short IDI**	**Incidence Δ Standard IDI**	**Δ-Regression β [95% CI]**	***p* (Delta reg.)**
UA pH < 7.10	635|216	6.9%	−0.5%	0.074 [0.026, 0.122]	0.002
UA pH < 7.00	635|216	0.6%	−2.3%	0.029 [0.008, 0.051]	0.008
Apgar 5 min < 7	635|216	1.4%	−0.9%	0.023 [−0.001, 0.048]	0.064
NICU admission	635|216	2.0%	0.9%	0.011 [−0.033, 0.056]	0.622
Metabolic acidosis	635|216	4.9%	−0.5%	0.053 [0.006, 0.100]	0.026
Composite neonatal adverse	635|216	6.8%	0.5%	0.063 [0.011, 0.116]	0.019
Postpartum anemia (Hb < 10, 24 h)	635|216	8.5%	4.6%	0.039 [−0.055, 0.132]	0.417
Postpartum hemorrhage	635|216	6.5%	4.6%	0.018 [−0.036, 0.073]	0.511
Blood transfusion	635|216	7.1%	2.8%	0.043 [−0.005, 0.091]	0.076
MCM (transfusion and/or ICU)	635|216	7.4%	2.8%	0.046 [−0.021, 0.114]	0.181
EPDS ≥ 13	379|137	19.3%	10.2%	0.090 [0.013, 0.167]	0.021
Uterine atony	635|216	4.9%	1.9%	0.030 [−0.013, 0.074]	0.171
Postpartum infection	635|216	1.3%	−0.9%	0.022 [−0.018, 0.062]	0.283
**(b) Continuous Outcomes**					
**Outcome**	***n* Pairs (Short|std)**	**Mean Δ Short IDI**	**Mean Δ Standard IDI**	**Δ-Regression β [95% CI]**	***p***
UA pH (continuous)	635|216	−0.018	−0.002	−0.016 [−0.032, 0.000]	0.051
UA lactate	635|216	0.21	−0.13	0.340 [0.063, 0.617]	0.016
UA base excess	635|216	−0.652	0.181	−0.834 [−1.449, −0.219]	0.008
Δ Hb 24 h (g/dL)	635|216	−0.100	−0.068	−0.031 [−0.221, 0.158]	0.745
Hb at 24 h (g/dL)	635|216	−0.199	−0.114	−0.085 [−0.297, 0.127]	0.433
EPDS total (continuous)	379|137	2.66	2.13	0.528 [−0.117, 1.174]	0.109
Apgar 5 min (continuous)	635|216	−0.087	0.111	−0.198 [−0.393, −0.002]	0.047

## Data Availability

The anonymized data supporting the findings of this study are available from the corresponding author upon reasonable scientific request and with approval from the institutional ethics committee. The data are not publicly available due to privacy and institutional data-governance restrictions under the Turkish Personal Data Protection Law (Law No. 6698, KVKK).

## Supplementary Materials

The following supporting information can be downloaded at: https://www.mdpi.com/article/10.3390/jcm15135053/s1.
