# Supplementary material for: Short Interdelivery Interval and Neonatal Acid–Base Status, Postpartum Anemia, and Postnatal Depression: A Retrospective Cohort Study with Within-Mother Sensitivity Analysis
Source: J Clin Med. 2026 Jun 29;15(13):5053. doi: 10.3390/jcm15135053 (PMC13362515; doi:10.3390/jcm15135053)
Supplement: Supplementary file 1 [file jcm-15-05053-s001.zip › jcm-4297901-supplementary-tables.pdf]

SUPPLEMENTARY TABLES

Short Interdelivery Interval and Neonatal Acid–Base Status, Postpartum Anemia, and Postnatal Depression: A Retrospective Cohort Study

Supplementary Table S1. Alternative IDI thresholds and restricted cubic spline (RCS) knot positions. Short-vs-standard IDI odds ratios (multivariable-adjusted, 95% CI) at five candidate cut-points (18, 21, 24, 27, 30 months) for the primary and key secondary outcomes. The 24-month threshold (WHO convention) is the pre-specified primary contrast. RCS knot locations (4 df) used in Figure 3 are listed in the lower sub-table.

(a) Adjusted OR [95% CI] at alternative thresholds

Outcome	<18 mo vs ≥18 mo	<21 mo vs ≥21 mo	<24 mo vs ≥24 mo	<27 mo vs ≥27 mo	<30 mo vs ≥30 mo
Neonatal acidosis (UA pH <7.10)	1.77 [1.09–2.88]	2.25 [1.30–3.88]	2.11 [1.07–4.13]	1.17 [0.55–2.46]	2.64 [0.51–13.70]
Postpartum anemia (Hb <10 g/dL, 24 h)	1.48 [1.12–1.96]	1.61 [1.22–2.12]	2.59 [1.86–3.61]	2.57 [1.65–3.98]	2.11 [1.08–4.13]
EPDS ≥13	1.82 [1.28–2.58]	1.86 [1.29–2.69]	2.19 [1.39–3.46]	1.67 [0.95–2.93]	5.07 [1.39–18.53]
Composite neonatal adverse	1.56 [0.99–2.46]	1.97 [1.19–3.23]	1.85 [1.01–3.39]	1.07 [0.54–2.12]	1.79 [0.49–6.55]
Strict MCM (transfusion and/or ICU)	1.62 [1.12–2.35]	1.68 [1.13–2.49]	1.53 [0.96–2.43]	1.20 [0.68–2.13]	0.87 [0.39–1.95]

(b) Restricted cubic spline knot positions (months)

Outcome	k1	k2	k3	k4
UA pH <7.10	12.2	17.4	22.0	29.9
Postpartum anemia (Hb <10)	12.2	17.4	22.0	29.9
EPDS ≥13	12.2	17.3	21.8	30.1
Composite neonatal adverse	12.2	17.4	22.0	29.9
Strict MCM	12.2	17.4	22.0	29.9

Supplementary Table S2. Operational definitions and data-source / measurement notes for every pre-specified primary, key secondary, and exploratory outcome used in the study. Preoperative anemia (covariate) uses the WHO threshold Hb <11 g/dL; postpartum anemia (primary outcome) uses the clinical-action threshold Hb <10 g/dL at 24 h.

Outcome	Operational definition	Notes
Neonatal acidosis (primary)	UA pH <7.10	Umbilical-artery gas pH at birth; threshold per Malin 2010 meta-analysis.
Maternal composite morbidity (MCM; primary)	Peripartum blood transfusion AND/OR maternal ICU admission	Strict MCM as pre-specified; distinct from CDC SMM and WHO near-miss.
Postpartum anemia (key secondary)	Hb <10 g/dL at 24 h postpartum	Clinical action threshold; triggers transfusion/iron protocol.
Postpartum depression (key secondary)	EPDS total ≥13 at postpartum day 10	Engindeniz 1996 Turkish validation; timing prespecified.
Composite neonatal adverse (key secondary)	Any of: UA pH <7.10, Apgar 5 min <7, NICU admission	Pre-specified composite; component-driven by UA pH (88% overlap). Sensitivity construct, not an independent endpoint.
UA pH <7.00 (exploratory)	UA pH <7.00	Severe metabolic compromise indicator.
Metabolic acidosis (exploratory)	UA pH <7.20 AND Base Excess ≤ −12 mmol/L	Composite biochemical criterion (ACOG Practice Bulletin 106).
Apgar 5 min <7 (exploratory)	Apgar score at 5 min <7	Operational cut-off for depressed newborn.
NICU admission (exploratory)	Admission to NICU within 24 h	Resource-based indicator; captures heterogeneous pathologies.
Postpartum hemorrhage (exploratory)	Clinician-adjudicated EBL >500 mL (vaginal) or >1000 mL (cesarean)	Obstetrician chart confirmation.
Blood transfusion (exploratory)	Any PRBC transfusion within 24 h of delivery	Peripartum period only.
Uterine atony (exploratory)	Clinician-documented atony requiring pharmacologic treatment	Excludes mild tone anomalies managed with massage only.
Postpartum infection (exploratory)	Endometritis, wound infection, or febrile morbidity 24 h–6 wk	Per chart-documented diagnosis codes.
Maternal ICU admission (exploratory)	Admission to adult ICU within 7 days of delivery	Any cause, peripartum.

Supplementary Table S3a. Variable-level missing-data summary at the index (second) delivery, N = 851 mothers. Missingness rates are reported overall and stratified by exposure group. Little's MCAR test is presented where applicable. EPDS missingness (20.9%) was the only non-trivial gap and was handled by MICE × 20 × 10 chained-equations imputation (Supplementary Table S4).

**Variable**	**Short IDI n missing (%)**	**Standard IDI n missing (%)**	**Overall n missing (%)**	**MCAR p**
Maternal age	0/635 (0.0%)	0/216 (0.0%)	0/851 (0.0%)	—
Gravidity	0/635 (0.0%)	0/216 (0.0%)	0/851 (0.0%)	—
Parity	0/635 (0.0%)	0/216 (0.0%)	0/851 (0.0%)	—
Gestational age	0/635 (0.0%)	0/216 (0.0%)	0/851 (0.0%)	—
Hb pre-op	0/635 (0.0%)	0/216 (0.0%)	0/851 (0.0%)	—
Hb 24 h	0/635 (0.0%)	0/216 (0.0%)	0/851 (0.0%)	—
UA pH	0/635 (0.0%)	0/216 (0.0%)	0/851 (0.0%)	—
UA pCO_2	0/635 (0.0%)	0/216 (0.0%)	0/851 (0.0%)	—
UA base excess	0/635 (0.0%)	0/216 (0.0%)	0/851 (0.0%)	—
UA lactate	0/635 (0.0%)	0/216 (0.0%)	0/851 (0.0%)	—
Apgar 5 min	0/635 (0.0%)	0/216 (0.0%)	0/851 (0.0%)	—
EPDS total	129/635 (20.3%)	49/216 (22.7%)	178/851 (20.9%)	0.520
Birth weight	0/635 (0.0%)	0/216 (0.0%)	0/851 (0.0%)	—
Birth length	0/635 (0.0%)	0/216 (0.0%)	0/851 (0.0%)	—

Supplementary Table S3b. EPDS availability comparison: baseline characteristics of participants with (n = 673) vs without (n = 178) observed EPDS scores at postpartum day 10. Variables compared with Mann–Whitney U (continuous) or χ^2 (categorical). Migrant status differs materially between the two groups (SMD −0.22, p = 0.009), indicating that EPDS missingness is not completely at random but plausibly MAR conditional on migrant status and language. This missingness-mechanism diagnostic motivates MICE × 20 chained-equations imputation (Table S4).

Variable	With EPDS (n = 673)	Without EPDS (n = 178)	SMD	p
Maternal age, mean ± SD	26.1 ± 4.7	26.3 ± 5.1	−0.03	0.976
Gestational age (weeks), mean ± SD	38.6 ± 1.3	38.5 ± 1.2	0.10	0.288
Parity, mean ± SD	2.7 ± 1.0	2.7 ± 0.9	−0.01	0.594
IDI (months), mean ± SD	20.1 ± 5.5	20.4 ± 5.5	−0.06	0.385
Pre-op hemoglobin (g/dL), mean ± SD	11.5 ± 0.7	11.4 ± 0.8	0.11	0.222
Short IDI (case), n (%)	506 (75.2)	129 (72.5)	0.06	0.520
Migrant status, n (%)	187 (27.8)	68 (38.2)	−0.22	0.009
Cesarean delivery (index), n (%)	262 (38.9)	81 (45.5)	−0.13	0.132
Induction of labor, n (%)	116 (17.2)	21 (11.8)	0.16	0.101
Prior cesarean, n (%)	237 (35.2)	64 (36.0)	−0.02	0.924
Postpartum anemia (Hb <10, 24 h), n (%)	310 (46.1)	77 (43.3)	0.06	0.560
UA pH <7.10, n (%)	51 (7.6)	20 (11.2)	−0.13	0.156
Apgar 5-min <7, n (%)	8 (1.2)	5 (2.8)	−0.12	0.221

Supplementary Table S4. Comparison of complete-case and MICE-pooled (m = 20 imputations, 10 iterations, miceforest random-forest chained equations) results for the four primary / key outcomes. Fraction of Missing Information (FMI) and Barnard–Rubin small-sample degrees-of-freedom correction are reported. Only EPDS ≥13 showed substantive change under MICE (OR 1.93 → 1.63); other outcomes were essentially fully observed.

(a) Complete-case vs MICE-pooled

Outcome	CC n	CC OR [95% CI]	CC p	MICE OR	MICE 95% CI	MICE p	FMI
Neonatal acidosis (UA pH <7.10)	851	2.37 [1.17–4.82]	0.017	2.37	1.17–4.82	0.017	0.00
Postpartum anemia (Hb <10, 24 h)	851	1.84 [1.26–2.68]	0.002	1.84	1.26–2.68	0.002	0.00
Postpartum depression (EPDS ≥13)	673	1.93 [1.20–3.10]	0.007	1.63	1.02–2.62	0.043	0.285
Composite neonatal adverse	851	2.06 [1.09–3.88]	0.026	2.06	1.09–3.88	0.026	0.00

(b) Imputation diagnostics (Barnard–Rubin df)

Outcome	m	β (pooled)	SE (pooled)	FMI	df (Barnard–Rubin)
Neonatal acidosis (UA pH <7.10)	20	0.865	0.363	0.00	∞
Postpartum anemia (Hb <10, 24 h)	20	0.611	0.190	0.00	∞
Postpartum depression (EPDS ≥13)	20	0.489	0.242	0.285	164.3
Composite neonatal adverse	20	0.723	0.322	0.00	∞

Supplementary Table S5. Covariate balance before and after inverse-probability-of-treatment weighting (IPTW). Standardized mean differences are shown pre- and post-weighting, along with weight-distribution diagnostics (min/max, 1st/99th-percentile trim, effective sample size).

(a) Covariate balance (|SMD|)

Covariate	SMD before IPTW	SMD after IPTW
Maternal age	−0.218	−0.010
Gravidity	0.094	0.031
Parity	0.014	0.024
Gestational age at delivery	−0.131	−0.037
Preoperative Hb	−0.512	0.030
Preoperative anemia (Hb <11)	0.345	−0.010

(b) Weight-distribution diagnostics

Diagnostic	Value
N used	851
Min stabilized weight	0.4866
Max stabilized weight	2.4668
Mean stabilized weight	0.9967
Weight trim (percentiles)	1st / 99th
P(Short IDI) marginal	0.746
Mean PS (treated / control)	0.765 / 0.692
Effective sample size (ESS)	812

Supplementary Table S6. Mediation analysis (VanderWeele & Vansteelandt difference method on the log-odds scale) with three candidate mediators of the Short-IDI → EPDS ≥13 pathway: postpartum 24-h anemia (Hb <10 g/dL), ΔHb (pre − 24 h), and 24-h absolute Hb. Total, natural direct (NDE) and natural indirect (NIE) effects are reported on the OR scale with 95% bias-corrected and accelerated (BCa) bootstrap intervals (10,000 resamples). Proportion mediated (PM) is on the log-OR scale.

**Mediator**	**n**	**Total OR [95% BCa]**	**NDE OR [95% CI]**	**NIE OR [95% CI]**	**Proportion mediated**
Postpartum anemia 24 h (Hb <10)	672	1.94 [1.24–3.23]	1.93 [1.25–3.21]	1.00 [0.94–1.07]	0.6% [−13.1%, +13.5%]
ΔHb (pre − 24 h), g/dL	672	1.94 [1.25–3.42]	1.98 [1.27–3.46]	0.98 [0.90–1.06]	−3.2% [−25.9%, +10.3%]
Hb 24 h (absolute, g/dL)	672	1.94 [1.22–3.25]	1.98 [1.23–3.34]	0.98 [0.88–1.05]	−3.2% [−30.0%, +10.9%]

Supplementary Table S7. Benjamini–Hochberg false-discovery-rate correction (BH-FDR) across the 13 pre-specified outcomes. Multivariable-adjusted primary ORs, raw p-values, q-values, and significance at q <0.05 are shown. Two outcomes (postpartum anemia and EPDS ≥13) remain FDR-positive; UA pH <7.10 is borderline (q = 0.075).

Outcome	MV-aOR [95% CI]	p (raw)	q (BH-FDR)	Significant (q <0.05)
Postpartum anemia (Hb <10, 24 h)	1.84 [1.26–2.68]	0.002	0.022	Yes
Postpartum depression (EPDS ≥13)	1.93 [1.20–3.10]	0.007	0.042	Yes
Neonatal acidosis (UA pH <7.10)	2.37 [1.17–4.82]	0.017	0.075	No (borderline)
Composite neonatal adverse	2.06 [1.09–3.88]	0.026	0.084	No (borderline)
UA pH <7.00	— (Firth IPTW: 5.61)	0.175	0.285	No
Metabolic acidosis	1.79 [0.89–3.60]	0.143	0.241	No
Apgar 5 min <7	1.65 [0.36–7.61]	0.416	0.505	No
NICU admission	1.12 [0.55–2.29]	0.699	0.669	No
Postpartum hemorrhage	1.12 [0.65–1.94]	0.759	0.770	No
Blood transfusion	1.88 [0.97–3.65]	0.059	0.256	No
Strict MCM	1.64 [0.85–3.16]	0.065	0.256	No
Uterine atony	1.41 [0.70–2.84]	0.504	0.505	No
Postpartum infection	1.72 [0.68–4.31]	0.199	0.412	No

Supplementary Table S8. E-values (VanderWeele & Ding, Ann Intern Med 2017) for the four primary / key outcomes plus strict MCM. For each outcome the adjusted OR is converted to an approximate RR using the baseline risk in the standard IDI group; the E-value (point estimate) and the E-value for the confidence-interval bound closest to the null are then computed. An E-value indicates the minimum strength of association, on the RR scale, that an unmeasured confounder would need to have with both exposure (short IDI) and outcome — above and beyond measured covariates — to fully explain away the observed association.

Outcome	MV-aOR	OR 95% CI	Baseline risk (std IDI)	E-value (point)	E-value (CI bound)
UA pH <7.10	2.37	1.17–4.82	0.046	3.88	1.58
Composite neonatal adverse	2.06	1.09–3.88	0.060	3.28	1.39
Postpartum depression (EPDS ≥13)	1.93	1.20–3.10	0.156	2.76	1.60
Strict MCM	1.64	0.85–3.16	0.116	2.55	1.00
Postpartum anemia (Hb <10, 24 h)	1.84	1.26–2.68	0.287	2.32	1.61

Footnote. E-values at the CI bound closest to the null fall to 1.39–1.61 for the primary outcomes, indicating residual sensitivity to unmeasured confounding at the interval limits. Baseline risk computed as the event proportion in the standard IDI group; RR approximation uses the formula $RR = OR / (1 - p_0 + p_0 \cdot OR)$.

Supplementary Table S9. Enrollment-quartile sensitivity analysis for the three primary outcomes. Because individual-level admission dates are not retained in the de-identified dataset, the cohort was split into four equal-sized quartiles based on within-group enrollment order as a proxy for the ~3-year (January 2023 – December 2025) calendar window. Adjusted ORs (aOR) for short IDI vs. standard IDI are reported within each quartile from the same primary multivariable logistic model (adjusted for maternal age, gestational age, parity, migrant status, cesarean delivery, induction of labor, and prior cesarean). Point estimates without confidence intervals reflect quasi-separation in sparse strata; direction is preserved but precision is reduced.

Outcome	Quartile	n	aOR [95% CI]	p	Note
Postpartum anemia (Hb <10, 24 h)	Q1 (earliest)	213	1.91 [0.97–3.76]	0.061	—
	Q2	213	3.30 [—]	—	CI not estimable
	Q3	212	4.10 [1.98–8.51]	<0.001	—
	Q4 (latest)	213	1.76 [0.91–3.40]	0.095	—
EPDS ≥ 13	Q1	172	3.05 [0.85–10.94]	0.086	—
	Q2	183	4.70 [1.34–16.50]	0.016	—
	Q3	160	0.91 [0.37–2.21]	0.832	—
	Q4	158	2.01 [—]	—	CI not estimable
UA pH <7.10	Q1	213	1.25 [—]	—	CI not estimable
	Q2	213	2.21 [—]	—	CI not estimable
	Q3	212	2.98 [0.62–14.20]	0.171	—
	Q4	213	4.45 [—]	—	CI not estimable

Footnote. Direction of effect for the anemia and EPDS outcomes is consistent (aOR > 1) across all four enrollment quartiles, supporting temporal stability of the primary findings. The UA pH < 7.10 outcome has <15 events in some quartiles, producing quasi-separation; point estimates remain above 1 but CIs are not estimable without penalization. Full calendar-quarter sensitivity (by admission date) is deferred pending re-identified date variables.

Supplementary Table S10. Variance inflation factors (VIF) for all covariates in the primary multivariable logistic model. VIF < 5 is considered acceptable; VIF ≥ 10 indicates serious multicollinearity. The only borderline values are cesarean delivery (current) and prior cesarean (VIF 5.10 and 5.14), reflecting the expected high correlation between index-delivery mode and delivery history. Sensitivity analyses with each of these covariates excluded (not shown) yielded aOR estimates that differed from the primary model by <5% for all three outcomes.

Covariate	VIF	Interpretation	Note
Maternal age	1.22	Acceptable	—
Gestational age (weeks)	1.17	Acceptable	—
Parity	1.20	Acceptable	—
Migrant status	1.06	Acceptable	—
Cesarean delivery (index)	5.10	Borderline	Shared variance with PriorCS
Induction of labor	1.15	Acceptable	—
Prior cesarean delivery	5.14	Borderline	Shared variance with CS index

Supplementary Table S11. Propensity-score model specification and coefficient estimates for the IPTW analyses (Section 2.6.5). A single logistic-regression propensity-score (PS) model for the exposure short interdelivery interval (IDI <24 months vs. ≥24 months) was fitted on all 851 women with complete data; the same PS was used for all six outcomes in Table 2 (UA pH <7.10, maternal composite morbidity, postpartum anemia, EPDS ≥13, composite neonatal adverse, blood transfusion). The PS formula was: short_IDI ~ maternal age + gravidity + parity + gestational age + pre-operative hemoglobin + pre-operative anemia. β coefficients are on the log-odds (logit) scale; ORs are exp(β). The six covariates were selected a priori on the basis of clinical relevance and the four imbalanced covariates identified in Table 1 (Section 3.1). No interaction or polynomial terms were specified.

(a) PS-model logistic regression coefficients

Covariate (description)	β (SE)	p	OR	95% CI (β)	95% CI (OR)
Intercept	+16.4785 (3.3150)	<0.001	14339224.240	9.981 to 22.976	21618.330–9511064030
Maternal age (years)	-0.0566 (0.0179)	0.002	0.945	-0.092 to -0.021	0.912–0.979
Gravidity (count)	+0.3311 (0.1666)	0.047	1.393	0.005 to 0.658	1.005–1.930
Parity (count)	-0.2432 (0.1889)	0.198	0.784	-0.613 to 0.127	0.541–1.135
Gestational age (weeks)	-0.1063 (0.0646)	0.100	0.899	-0.233 to 0.020	0.792–1.021
Pre-operative hemoglobin (g/dL)	-0.8672 (0.1759)	<0.001	0.420	-1.212 to -0.522	0.298–0.593
Pre-operative anemia (Hb <11 g/dL, 0/1)	-0.3460 (0.3150)	0.272	0.708	-0.964 to 0.272	0.382–1.312

Model fit: n = 851; log-likelihood = −452.17; McFadden pseudo-R² = 0.062; AIC = 918.34; BIC = 951.56; LR test χ² = 59.83, p < 0.001.

(b) IPTW reproduction check — manuscript Table 2 OR values

Outcome	N	Manuscript Table 2 OR	Reproduced OR (95% CI)	Δ from reported	Match
UA pH <7.10	851	2.25	2.248 (1.117–4.525)	0.002	Exact
Maternal composite morbidity	851	1.35	1.353 (0.849–2.158)	0.003	Exact
Postpartum anemia (Hb <10, 24 h)	851	1.86	1.859 (1.288–2.683)	0.001	Exact
EPDS ≥13 (day 10)	673	1.78	1.784 (1.143–2.784)	0.004	Exact
Composite neonatal adverse	851	1.89	1.886 (1.021–3.482)	0.004	Exact
Blood transfusion	851	1.83	1.834 (0.976–3.447)	0.004	Exact

Footnote. *Continuous covariates: maternal age (years), gravidity (count), parity (count), gestational age (weeks), pre-operative hemoglobin (g/dL). Pre-operative anemia is a binary indicator (1 if Hb <11 g/dL on admission for the index delivery, 0 otherwise). Missing values for covariates were median-imputed within the PS-fitting step (<1% missingness for all covariates). A single-PS approach was chosen a priori to maintain comparability of weights across the four outcome domains. IPTW OR values reproduce manuscript Table 2 column J to two decimal places for all six outcomes (Δ < 0.005).*

Supplementary Table S12. IPTW weight diagnostics, pre- and post-weighting covariate balance, and propensity-score distribution and overlap (single PS model, Supplementary Table S11). This table extends Supplementary Table S5 by providing the full distributional summary of the propensity score and additional weight-diagnostic statistics; the standardized mean differences (SMDs) and weight summary in Panels (a) and (b) are identical to those in Supplementary Table S5. Stabilized weights were computed as $sw = p/\pi$ for short-IDI and $(1 - p)/(1 - \pi)$ for standard-IDI, where p is the marginal probability of short IDI (0.7462) and π is the predicted PS. Weights were truncated at the 1st and 99th percentiles.

(a) Stabilized-weight diagnostics

Statistic	Value
N (total)	851
P(short IDI) marginal	0.7462
1st-percentile cutoff (weight)	0.4866
99th-percentile cutoff (weight)	2.4668
# truncated (low / high / total)	9 / 9 / 18 (2.12%)
Min weight (after truncation)	0.4866
Max weight (after truncation)	2.4668
Mean ± SD weight	0.9967 ± 0.2837
Median weight	0.9460
Effective sample size (Kish)	787.30 (92.51% of N)

(b) Standardized mean differences (SMDs), pre- and post-IPTW

Covariate	|SMD| pre-IPTW	|SMD| post-IPTW	Direction (pre)	Balance achieved (<0.10)?
Maternal age (years)	0.218	0.010	−	Yes
Gravidity (count)	0.094	0.031	+	Yes
Parity (count)	0.014	0.024	+	Yes
Gestational age (weeks)	0.131	0.037	−	Yes
Pre-operative hemoglobin (g/dL)	0.512	0.030	−	Yes
Pre-operative anemia (Hb <11)	0.345	0.010	+	Yes

(c) Propensity-score distribution and overlap

Group	Min	p5	p25	Median	p75	p95	Max
Short IDI (treated, n=635)	0.2957	0.5638	0.6961	0.7836	0.8449	0.9089	0.9520
Standard IDI (control, n=216)	0.3839	0.4988	0.6107	0.6997	0.7905	0.8770	0.9471
Common support range: [0.3839, 0.9471]. Positivity violations: 3 subjects (retained after weight truncation at 1st/99th percentiles).							

Footnote. *Balance threshold |SMD| < 0.10 is the conventional cutoff for adequate covariate balance (Austin 2009). Pre-operative hemoglobin had the largest pre-weight imbalance (|SMD| = 0.512) and was substantially attenuated after IPTW (|SMD| = 0.030). All six covariates achieved post-IPTW |SMD| < 0.10. Common support is the*

intersection of PS ranges between the two groups; the three subjects whose PS values fell outside this intersection were retained in the IPTW analysis after weight truncation at the 1st/99th percentiles. Effective sample size (Kish, after truncation) is 787.30 (92.5% of N = 851), indicating that the precision loss due to IPTW is modest. The Supplementary Table S5 ESS value (n = 812) refers to the pre-truncation Kish estimate.

Supplementary Table S13. Restricted-cubic-spline (RCS) knot-placement sensitivity analysis for the four primary and key secondary outcomes (Section 2.6.8). Four alternative knot configurations were fitted using Harrell-style restricted cubic splines, with the reference fixed at IDI = 24 months. Adjustment set per outcome panel: Panels A, C, D — maternal age, gravidity, parity, gestational age, pre-operative hemoglobin, pre-operative anemia; Panel B (postpartum anemia primary) — same minus pre-operative anemia by design (collider with respect to the primary endpoint, Section 2.6.5). Adjusted ORs and 95% Wald confidence intervals at IDI = 12, 18, and 30 months are reported relative to the reference IDI = 24 months. The manuscript's primary specification is C2 (4 knots at the 5th, 35th, 65th, and 95th percentiles of the IDI distribution, df = 3).

Configuration	Knots (months)	df	OR @12 mo [95% CI]	OR @18 mo [95% CI]	OR @30 mo [95% CI]
(a) Panel A — UA pH <7.10 (N = 851)					
C1: 3 knots (10/50/90 pct)	13.1, 19.8, 28.0	2	1.84 [0.90–3.79]	1.45 [1.05–2.01]	0.63 [0.31–1.26]
**C2: 4 knots (5/35/65/95 pct) (PRIMARY)**	12.2, 17.4, 22.0, 29.9	3	1.94 [0.91–4.15]	1.89 [0.96–3.72]	0.86 [0.34–2.14]
C3: 4 knots (20/40/60/80 pct)	14.8, 18.1, 21.3, 25.1	3	1.87 [0.89–3.89]	1.93 [0.96–3.89]	0.79 [0.33–1.90]
C4: 5 knots (5/27.5/50/72.5/95)	12.2, 16.1, 19.8, 23.4, 29.9	4	3.39 [1.36–8.47]	2.99 [1.30–6.87]	1.29 [0.53–3.15]
(b) Panel B — Postpartum anemia (Hb <10 g/dL at 24 h) (N = 851)					
C1: 3 knots (10/50/90 pct)	13.1, 19.8, 28.0	2	1.02 [0.63–1.64]	1.26 [1.05–1.51]	0.57 [0.39–0.83]
**C2: 4 knots (5/35/65/95 pct) (PRIMARY)**	12.2, 17.4, 22.0, 29.9	3	1.01 [0.62–1.65]	1.08 [0.72–1.63]	0.50 [0.30–0.82]
C3: 4 knots (20/40/60/80 pct)	14.8, 18.1, 21.3, 25.1	3	1.04 [0.65–1.66]	1.08 [0.70–1.66]	0.51 [0.32–0.81]
C4: 5 knots (5/27.5/50/72.5/95)	12.2, 16.1, 19.8, 23.4, 29.9	4	1.06 [0.60–1.87]	1.12 [0.72–1.75]	0.51 [0.30–0.88]
(c) Panel C — Postnatal depression (EPDS ≥13 at day 10) (N = 673)					
C1: 3 knots (10/50/90 pct)	13.1, 19.7, 28.0	2	1.47 [0.86–2.51]	1.43 [1.14–1.80]	0.55 [0.34–0.89]
**C2: 4 knots (5/35/65/95 pct) (PRIMARY)**	12.2, 17.3, 21.8, 30.1	3	1.47 [0.84–2.55]	1.76 [1.10–2.83]	0.68 [0.36–1.28]
C3: 4 knots (20/40/60/80 pct)	14.8, 18.0, 21.0, 25.2	3	1.50 [0.88–2.56]	1.87 [1.15–3.06]	0.70 [0.39–1.27]
C4: 5 knots (5/27.5/50/72.5/95)	12.2, 16.1, 19.7, 23.3, 30.1	4	1.36 [0.71–2.61]	1.63 [0.95–2.80]	0.63 [0.31–1.30]
(d) Panel D — Composite neonatal adverse (N = 851)					
C1: 3 knots (10/50/90 pct)	13.1, 19.8, 28.0	2	1.81 [0.90–3.66]	1.48 [1.07–2.04]	0.58 [0.29–1.17]
**C2: 4 knots (5/35/65/95 pct) (PRIMARY)**	12.2, 17.4, 22.0, 29.9	3	1.90 [0.91–3.98]	1.92 [1.00–3.69]	0.81 [0.33–1.98]
C3: 4 knots (20/40/60/80 pct)	14.8, 18.1, 21.3, 25.1	3	1.84 [0.90–3.77]	2.03 [1.03–4.00]	0.76 [0.32–1.80]

C4: 5 knots (5/27.5/50/72.5/95)	12.2, 16.1, 19.8, 23.4, 29.9	4	3.39 [1.39–8.25]	3.05 [1.36–6.82]	1.23 [0.51–2.95]

Footnote. *Across the four configurations, the OR point estimates at IDI = 18 months remain in the 1.05–3.05 range across the four panels, with overlapping confidence intervals. The 5-knot specification (C4) yields the highest point estimates and the widest confidence intervals, consistent with overfitting at* $n \leq 851$*. Configurations C1, C2, and C3 produce qualitatively concordant results, supporting the robustness of the manuscript's primary 4-df, 5/35/65/95-percentile specification (C2).*
