# Supplementary figures and images for: Short Interdelivery Interval and Neonatal Acid–Base Status, Postpartum Anemia, and Postnatal Depression: A Retrospective Cohort Study with Within-Mother Sensitivity Analysis
Source: J Clin Med. 2026 Jun 29;15(13):5053. doi: 10.3390/jcm15135053 (PMC13362515; doi:10.3390/jcm15135053)

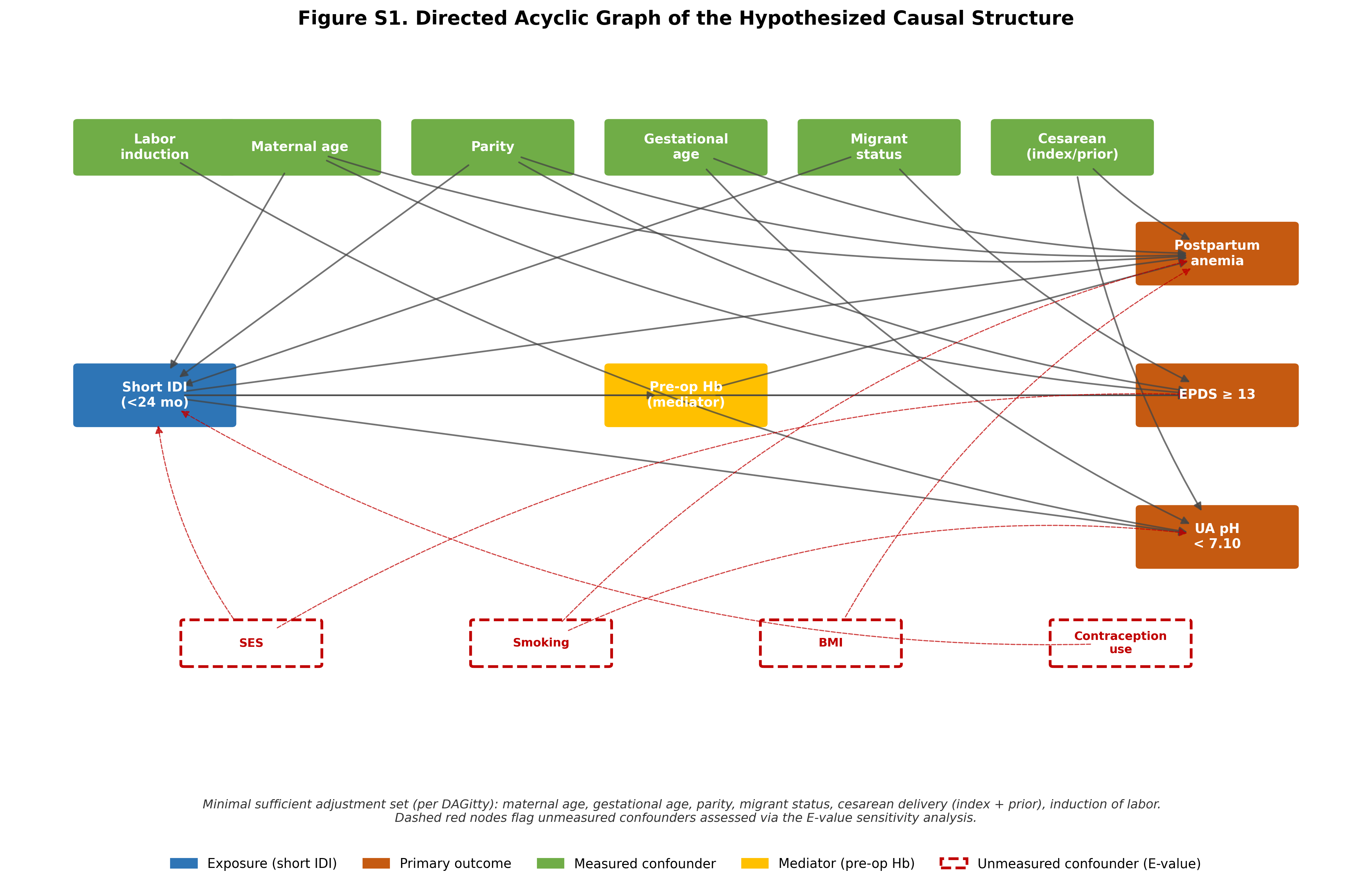

Supplement: Supplementary file 1 [file jcm-15-05053-s001.zip › FigureS1_DAG.png]

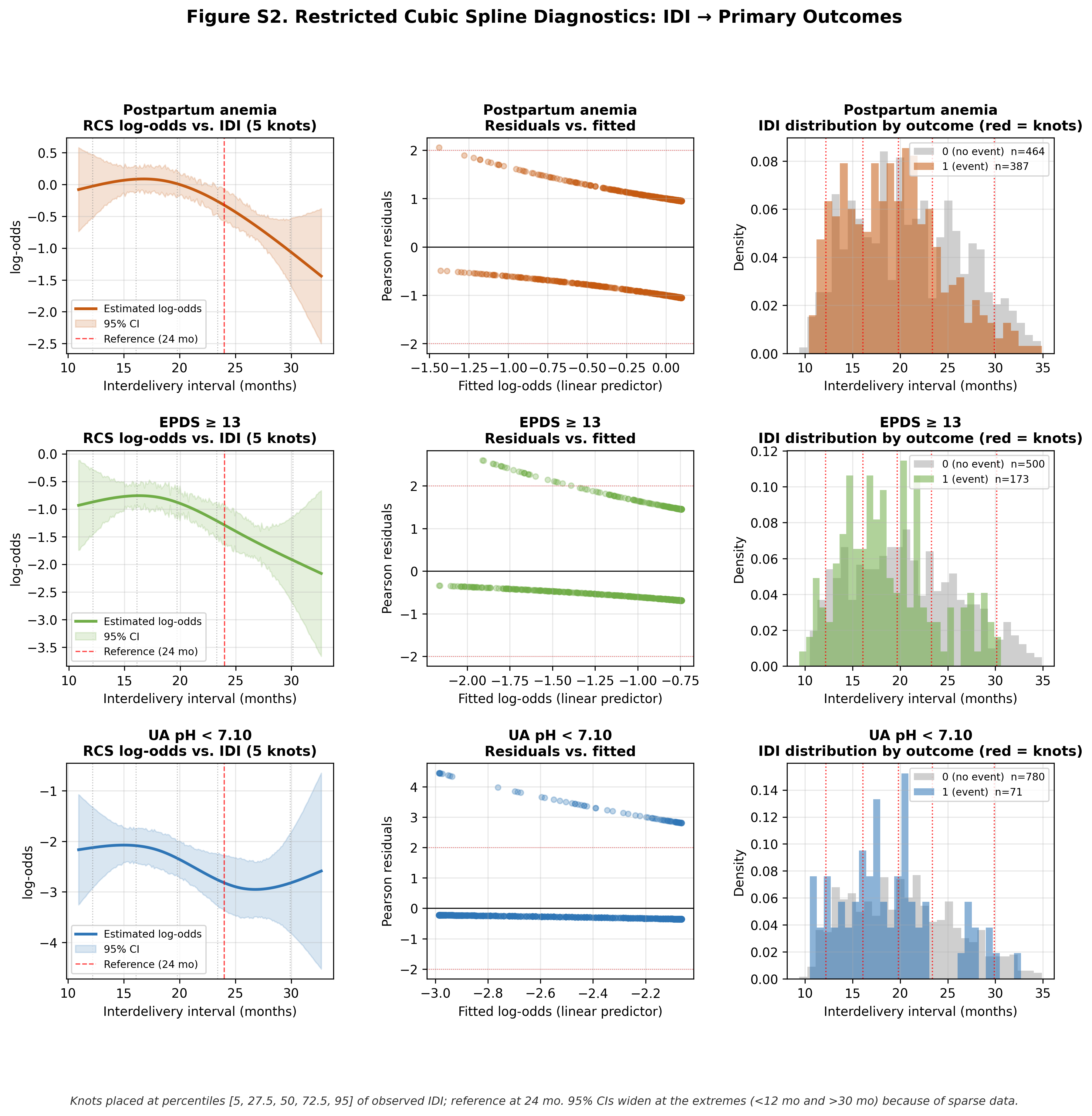

Supplement: Supplementary file 1 [file jcm-15-05053-s001.zip › FigureS2_RCS_Diagnostics.png]
